# Validation of rice genome sequence by optical mapping

**DOI:** 10.1186/1471-2164-8-278

**Published:** 2007-08-15

**Authors:** Shiguo Zhou, Michael C Bechner, Michael Place, Chris P Churas, Louise Pape, Sally A Leong, Rod Runnheim, Dan K Forrest, Steve Goldstein, Miron Livny, David C Schwartz

**Affiliations:** 1 Laboratory for Molecular and Computational Genomics, University of Wisconsin-Madison, UW Biotechnology Centre, 425 Henry Mall, Madison, Wisconsin 53706, USA; 2 Department of Chemistry, Laboratory of Genetics; University of Wisconsin-Madison, Madison, Wisconsin 53706, USA; 3 Laboratory of Genetics; University of Wisconsin-Madison, Madison, Wisconsin 53706, USA; 4 USDA-ARS, CCRU, Department of Plant Pathology, University of Wisconsin-Madison, Madison, Wisconsin 53706, USA; 5 Department of Computer Sciences, University of Wisconsin-Madison, Madison, Wisconsin 53706, USA

## Abstract

**Background:**

Rice feeds much of the world, and possesses the simplest genome analyzed to date within the grass family, making it an economically relevant model system for other cereal crops. Although the rice genome is sequenced, validation and gap closing efforts require purely independent means for accurate finishing of sequence build data.

**Results:**

To facilitate ongoing sequencing finishing and validation efforts, we have constructed a whole-genome SwaI optical restriction map of the rice genome. The physical map consists of 14 contigs, covering 12 chromosomes, with a total genome size of 382.17 Mb; this value is about 11% smaller than original estimates. 9 of the 14 optical map contigs are without gaps, covering chromosomes 1, 2, 3, 4, 5, 7, 8 10, and 12 in their entirety – including centromeres and telomeres. Alignments between optical and *in silico *restriction maps constructed from IRGSP (International Rice Genome Sequencing Project) and TIGR (The Institute for Genomic Research) genome sequence sources are comprehensive and informative, evidenced by map coverage across virtually all published gaps, discovery of new ones, and characterization of sequence misassemblies; all totalling ~14 Mb. Furthermore, since optical maps are ordered restriction maps, identified discordances are pinpointed on a reliable physical scaffold providing an independent resource for closure of gaps and rectification of misassemblies.

**Conclusion:**

Analysis of sequence and optical mapping data effectively validates genome sequence assemblies constructed from large, repeat-rich genomes. Given this conclusion we envision new applications of such single molecule analysis that will merge advantages offered by high-resolution optical maps with inexpensive, but short sequence reads generated by emerging sequencing platforms. Lastly, map construction techniques presented here points the way to new types of comparative genome analysis that would focus on discernment of structural differences revealed by optical maps constructed from a broad range of rice subspecies and varieties.

## Background

Several genome centres have established very effective "pipelines" for the sequencing of entire genomes, often using a mixed strategy drawing data from mapped clones and whole genome shotgun efforts [1]. However, such pipelines are rapidly evolving through the incorporation of new, high-throughput sequencing platforms, that obviate conventional clone libraries, or Sanger sequencing chemistries [2,3], but yield sequence reads of limited length. As such, current and emerging sequencing strategies benefit from the availability of compatible physical maps guiding clone selection, or facilitating assemblies that may also enhance validation efforts [1,3,4]. Furthermore, physical maps become a critical necessity when spanning repeat-rich genomic regions, typified by telomeric or centromeric portions of chromosomes. Although clone fingerprint [5] or end-sequence maps [6] are widely used in whole-genome squencing efforts, gaps and sequence assembly errors may persist stemming from "clone drop-outs," or uncertainties in the map assembly process caused by the presence of repeated sequence elements. Clearly, physical maps will continue to be an important feature of large-scale sequencing projects, but new mapping approaches must advance in ways that effectively deal with the trend towards obviation of traditional libraries and the abundance of modestly sized reads. The main issues will likely center on the validation of "strategically" unfinished genome sequences and comprehensive description of genome structure. These issues become acute when sequenced genomes are selected from nascently described organisms lacking genetic resources or an associated scientific community. Consequently, future comparative studies could suffer from sequencing errors, and not fully discern structural variation – a major feature of genome evolution and a source of disease genotypes.

The rice (*Oryza sativa*) genome was originally chosen for sequencing because it is a staple food crop for more than half of the world's population, in addition to its many genetic attributes, or resources that include: a compact genome (~400 Mb), well-defined genetic maps, Yeast Artificial Chromosome (YAC) and Bacterial Artificial Chromosome (BAC)/P1-derived Artificial Chromosome (PAC) map resources, comprehensive sequence-tagged or transcript maps, and efficient genetic transformation techniques [7-20]. Rice also shares extensive syntenic relationships with other cereal plants bearing huge genome sizes such as maize (~2,500 Mb), barley (~4,900 Mb), and wheat (~15,000 Mb) [21-26]. The large-scale sequencing of rice (*O. sativa *ssp. *japonica *cv Nipponbare) was initiated in 1998 under the auspices of the International Rice Genome Sequencing Project (IRGSP), with joint efforts from Japan, the United Sates, China, Brazil, Great Britain, France, India, Korea, and Thailand [27]. IRGSP members decided at the time to pursue the collaboration-friendly, clone-by-clone, or BAC/PAC-by-BAC/PAC strategy supported by extensive map resources. For example, BAC/PAC draft sequences or contigs were anchored and oriented on the rice genetic maps – these contigs were further augmented by BAC-end sequencing (*via *Sequence-Tagged Connectors) and contig-end walking. BAC maps and fibre Fluorescent *in situ *Hybridization (FISH) were also used for characterization of gaps present within low recombination regions or genomic portions showing modest BAC/PAC coverage [17,19,28,29]. The IRGSP release of the rice genome is now finished with publication of the analysis and annotation of these data [29-35]. Here, the IRGSP sequence for each chromosome – Build 4.0, released in August, 2005 – is represented as a "pseudomolecule," or a virtual contig. Each pseudomolecule is constructed by joining PAC/BAC sequences according to their order determined by comparison with a previously constructed physical map [30]. Finishing steps include identification and removal of overlapping sequences with resulting physical gaps replaced by a variable number of successive "N's", reflecting their estimated breadth. There are 62 physical gaps including 17 telomeric gaps, and 9 centromeric gaps with a total size of ~18.1 Mb [30], with one gap closed in chromosome 1 and some of them partially filled [36] within the current build.

In a parallel effort, TIGR (The Institute for Genomic Research) also constructed similar pseudomolecules for each of 12 rice chromosomes [37] to enlarge the span of regions comprising blocks of contiguous sequence – their approach included: the resolution of discrepancies between overlapping BAC/PAC clones, trimming of overlap regions, and linking of unique sequences. These efforts relied on 3,450 rice BAC/PAC clone sequences obtained from the IRGSP; of these, 3,408 BAC/PAC clones (98.8%) were finished, and 42 BAC/PAC (1.2%) clones were unfinished (phase 2), as defined by Genbank. As such, the data show many gaps between clones, *i.e*., physical gaps, denoted by "1000N's" in the final pseudomolecules. Finally, centromeres were identified using the "CentO" centromeric sequence [38]. There are 48 physical gaps within the 12 pseudomolecules including gaps at 10 centromeres.

In addition to the rice genome sequence from the IRGSP and TIGR, the draft rice genome sequence of the same cultivar Nipponbare was generated by two separate private sources: Pharmacia, Inc. (previously part of Monsanto, Inc., Peapack, NJ) and Syngenta, Inc. (San Diego, CA) [39]. A 259 Mb draft sequence from Pharmacia was also generated by a clone-by-clone based strategy [40], while the 390 Mb Syngenta rice genome draft sequence consisting of 42,109 sequence contigs was obtained using a whole-genome shotgun sequencing approach with an estimation of 32,000 to 50,000 genes for this cultivar. A draft sequence (361 Mb out of the estimated genome size of 466 Mb) of the *O. sativa *L. ssp. *indica *cultivar (93-11) was also obtained by the Beijing Genomics Institute (BGI) [41] using a whole-genome shotgun sequencing approach with an estimated gene count of 46,022 to 55,615 for this subspecies. Using TIGR's pseudomolecules and publicly available rice EST sequence data, Affymetrix has recently constructed a rice gene expression array with 46,115 rice gene models (Affymetrix, personal communication). In this regard, amongst sequenced genomes rice comprises the largest number of predicted genes, with more genes than human, and almost double that of *Arabidopsis thaliana*.

Although the current releases of rice sequence from IRGSP, and TIGR are of very high quality, difficult gaps remain to be spanned for each of the 12 chromosomal pseudomolecules. These gaps persist because some reside within genomic regions showing sparse coverage of genetic markers used for anchoring BAC/PAC clones, and others suffer from library construction, which may bias against heavily repetitive regions. Importantly, existing gaps probably contain many functional genes [36], even within centromeric regions [42], in addition to information describing chromosomal structure. Furthermore, sequence contigs, within the pseudomeolecules, may still contain errors, in part, arising from assemblies conducted in repeat-rich regions of the rice genome. Given these issues, we constructed a genome wide optical map for determination of the size of sequence gaps and for identification of problematic sequence assemblies (discordances) through the analysis of sequence build alignments against our optical restriction map.

Optical mapping is now a robust, automated system for the construction of whole genome ordered restriction maps from ensembles of individual genomic DNA molecules [43-55]. Library construction, PCR amplification, hybridization, and their attending artefacts are obviated in optical mapping, since genomic DNA is the analyte and restriction enzymes are used to generate reliable markers. Therefore, high-resolution physical maps are created on a whole genome basis presenting an organism's genetic constitution in a form directly linkable to sequence data. Using a newly automated version of optical mapping system, we have constructed a whole genome physical map for the rice genome using the optical mapping system, employing schemes akin to whole genome shotgun sequencing approaches. Given the results presented here, we find that there is no practical limit to the optical mapping of large, complex genomes, even in the absence of any sequence information, since the level of automation we have achieved provides ample data sets for our assembly techniques to span entire genomes. However, accessible sequence data for any genome provides direct links to a multitude of annotation and analysis tools that are especially facilitated when genomes are chosen to be strategically sequenced, such as maize.

More specifically, we present the construction of a whole-genome shotgun optical restriction map, using SwaI, of the rice (*Oryza sativa*) genome, and its comparison to sequence builds. Because physical distances (in kb) between restriction sites are accurately determined by optical mapping, alignments of an "*in silico" *restriction map, constructed from sequence data, against our optical map reveal discordances characterized by features commonly associated with ordered restriction maps. They include: missing or extra restriction "cuts," missing or extra restriction fragments, and significant alterations of restriction fragment sizes or patterns. Also, large-scale discordances covering hundreds of kilobases are discoverable and described here.

As such, we show that a high-resolution physical map based on the direct analysis of genomic DNA, spans existing sequence physical gaps, validates the genome sequence assembly, characterizes gaps, corrects sequence misassemblies, and creates a physical scaffold for sequence finishing. We expect that this map will also secure a resource for the genome sequencing communities at-large in their investigation of rice subspecies and cultivars. We also think that the maps presented here will facilitate the final validation of the rice sequence data, which should strengthen the important role that this genome is already playing as an accessible model system for other plants and cereal crops.

## Results

### Data acquisition and map assembly

**Figure 1:**
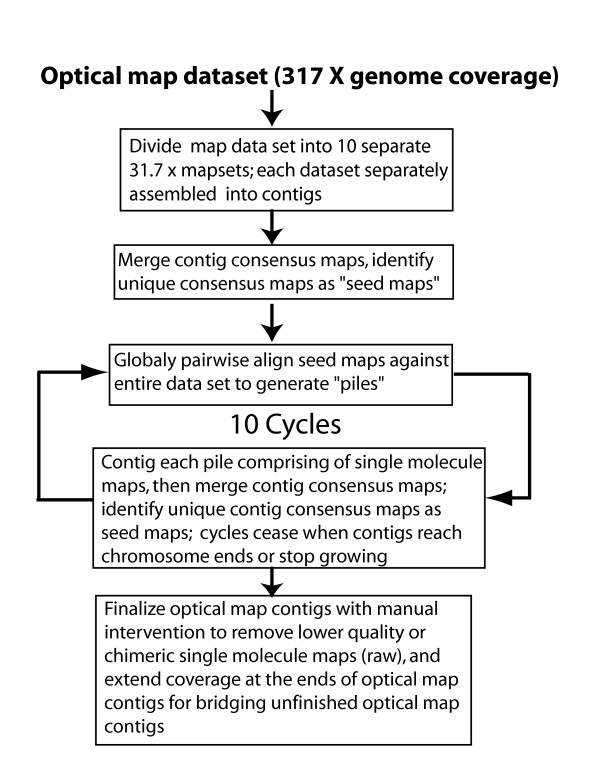
Flow chart showing the strategy used for the assembly of optical maps.

The whole-genome shotgun optical mapping approach [44,55] was used for the construction of SwaI restriction maps covering about 97% of the ~389 Mb rice genome. A total of 260,205 DNA molecules, ranging in size from 300 kb to 3600 kb, were individually mapped (Methods), representing 123,341 Mb in mass, or about 317 X coverage of the rice genome. The average size of molecules in this raw data set is 474.02 kb with an average restriction fragment size of 20.79 kb. Although this value is significantly larger than the average restriction fragment size calculated from sequence data (13.89 kb; fragments less than 0.5 kb are merged with neighbouring fragments), our assembly scheme (Fig. 1) segregates well-digested molecules for their inclusion into the final map contig assemblies. In total, there are 29,445 single DNA molecule maps (14,569 Mb) composing the finished contigs, making the rate of contig formation 11.8%.

Because the rice genome is significantly larger than previous genomes we have optically mapped, and assembly of optical maps is computationally demanding, we designed our assembly scheme (Fig. 1) to effectively leverage cluster computing resources for handling very large single molecule data sets. In this way, map assembly software utilizes advantages offered by cluster computing techniques [56] (Methods) for full *de novo *map assembly. Although, we could more easily use available sequence as a scaffold for facilitating map assembly, we reasoned that a purely *de novo *map assembly approach would obviate any concerns, regarding potential bias from nascent map alignments guided by sequence data. As such, our scheme embodies two major stages:

#### 1) Generation of "seed" or consensus maps

Here, we divide the entire rice optical map data set into 10 equally sized bins for their independent assembly into provisional contigs that serve as "seeds" for augmentation and growth into larger, more confident map scaffolds. Our initial procedure does not construct finished chromosome contigs, but does break the very large map data set into parallel, computationally manageable portions easily handled by the computer memory requirements of the map assembler [57-59]. The assembly step used for each bin produces a group of "consensus maps," or restriction maps comprising all significant restriction enzyme cleavage sites found within their respective contigs. The removal of redundant maps and the merging of overlapping consensus maps foster their subsequent utility.

#### 2) Growing contigs from seed maps

The previous step generates a set of seed maps. This process also culls high-quality optical maps from very large data sets; however, for completeness, nascent contigs (195) must be joined and merged for spanning entire chromosomes. We accomplish this through pairwise alignment of the complete map data set against the entire collection of seed maps. This operation accumulates "piles" of independently aligned single molecule maps that are then isolated from their seed map scaffolds and separately assembled into contigs using the map assembler. The previous step produces an updated generation of seed maps that are merged for identification of redundant maps, which are then removed. These assemblies are augmented through 10 iterations of the contig growing process.

After such iterations, the number of unique optical map contigs dropped from 195 to 29 with an average contig size of 13.57 Mb spanning 393.62 Mb. At this stage, joining operations deal with ends of contigs that result from issues specific to optical mapping. Here, contigs produce gaps within genomic regions like centromeres with a low density of SwaI restriction sites in rice genome, or through incorporation of low quality maps stemming from chimeric molecules (imaging may falsely merge several molecules). Therefore manual intervention removes low quality maps for restarting the contig growing process. Manual steps that test gaps for potential growth also ensure proper placement of *bona fide *contig ends within telomeric regions. Such manual steps were then validated by disassembly of map contigs into their original collection of maps, followed by new assemblies and joining operations.

**Figure 2:**
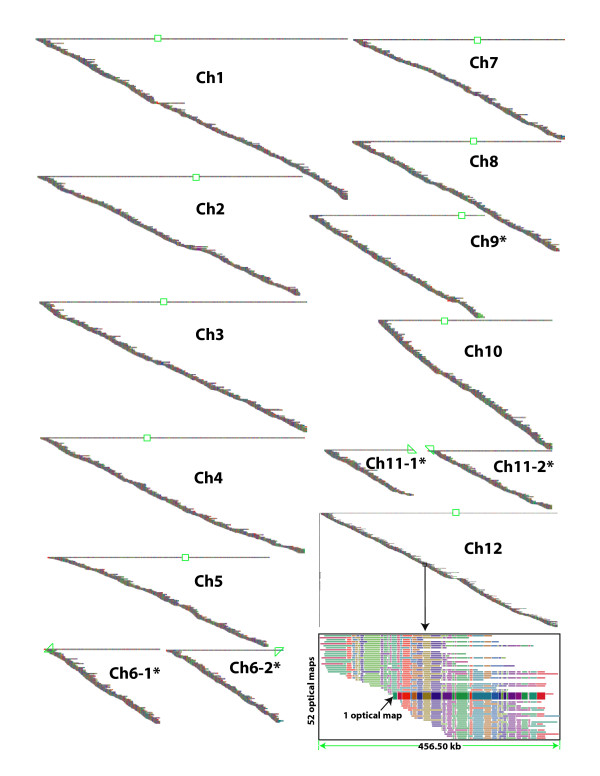
A whole-genome optical map of rice (*Oryza sativa *ssp. *japonica *cv. Nipponbare). The 14 optical map contigs are displayed as horizontal lines representing consensus maps; their centromeric regions located by green boxes and partial boxes indicate incomplete centromeric coverage. A consensus map comprises many (29,512 maps; 14 contigs) individual restriction maps, each constructed from one (~470 kb) endonuclease digested molecule shown overlapping other molecules along the accompanying diagonal track. Chromosomes marked with an "*" indicate partial optical map contigs. Inset shows a zoomed view of a ~400 kb interval on chromosome 12 (28.19 Mb). Here, each horizontal track depicts an optical map; its "daughter" restriction fragments are consecutive colored bars and congruent fragments across separate optical maps are color-keyed. Since restriction digestion is not quantitative, some bars (restriction fragments) bear missing or false cleavage sites – relative to the consensus map – flagged by disparate colors.

**Figure 4:**
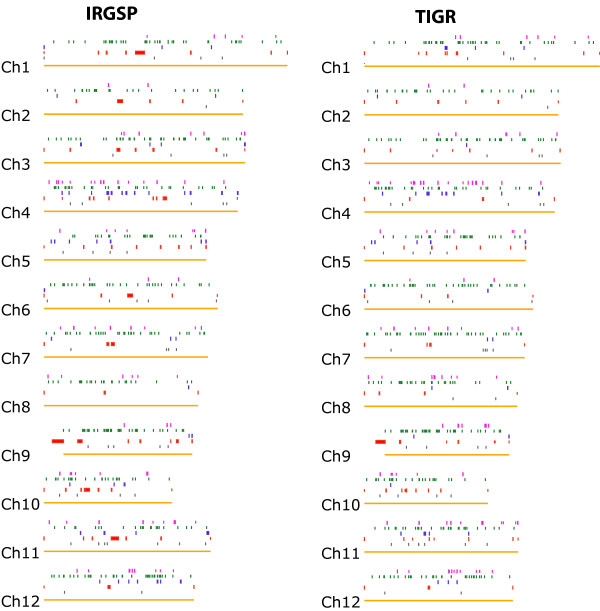
Whole genome view showing optical map *vs. *IRGSP or TIGR sequence data (pseudomolecules) – identification of errors and their loci (Additional file 1 and 2). 6 tracks depict data and comparison for each of the rice chromosomes (1–12): **track 1 **(gold solid horizontal line), *in silico *SwaI maps of the pseudomolecule data; **track 2 **(grey bars), false cut – cut present in optical map, but absent in sequence data; **track 3 **(red bars), gaps present in sequence but filled by optical maps; **track 4 **(blue bars), sequence misassemblies; **track 5 **(green bars), missing cut – cut present in sequence, but absent in map data; **track 6 **(magenta bars), new gaps called within the sequence pseudomolecule by optical maps (Table 2 and 3).

Using this scheme, 14 optical consensus maps were assembled having a total mass of 378.31 Mb. Of these, there were 9 finished optical map contigs representing chromosome 1, 2, 3, 4, 5, 7, 8, 10, and 12, identified based on the comparison between the consensus maps and the *in silico *maps from the IRGSP pseudomolecules. Although 14 optical map contigs were produced, 9 out of the 12 chromosomes reached completion (1, 2, 3, 4, 5, 7, 8, 10 and 12; Figs. 2 &4). Aside from the lack of gaps within these maps, we consider them finished because each map contig shows more than 5 single molecule maps defining each of the two blunt ends (absence of significant map "overhangs") – these sharply demarcated contig ends likely represent telomeric ends. The remaining 3 chromosomes have gaps occurring within centromeres (Ch 6 and Ch 11) – showing two blunt ends within telomeric regions – and a blunt-ended contig (Ch 9) spanning a telomeric region on the long arm, but not fully covering the short arm. Here lies the nucleolar organizer harboring ribosomal repeats having a low density of SwaI restriction sites that prevent joining operations.

### Genome-wide comparisons between optical and other physical maps

There are four genome-wide physical maps available for the rice genome and salient findings concerning chromosomal sizes are compared to optical mapping data in Table 1. The first is a molecular linkage map or genetic map with 3,267 RFLP or cDNA clone probes [20,60]. The second is a YAC-based physical map based on 2,275 DNA markers from the rice genetic map used to select 1,892 YACs from a library to generate 297 YAC contigs, and 142 YAC islands – it uniquely spans 270 Mb [7,20]. The third is a whole genome transcript map with 6,591 mapped EST markers which greatly helped to position the PAC/BAC clones throughout the rice genome [8]. The fourth is the sequence-ready BAC/PAC physical map, and these BAC/PACs were anchored and oriented on the genetic map which forged a minimum tiling path of BAC/PAC clones for each of the 12 rice chromosomes – it comprises 3,466 BAC/PAC clones showing 46 gaps [8].

**Table 1:** Rice (*Oryza sativa *ssp. *japonica *cv. Nipponbare) whole-genome shotgun optical mapping

**Ch.**	**Est. Size (Mb) YAC^a^**	**Est. Size (Mb) BAC^b^**	**FISH^c ^Size(Mb)/AR**	**IRGSP^d^**	**Opt. Map Contig (status)**	**Opt. Map (Mb)**	**Predicted Size (Mb)^e^/ArmRatio**	**Difference (%) YAC/BAC/FISH/IRGSP**	**Ave. frag. size (kb)**	**# of Mol.**	**X Cov.**	**Ave. frag. size SD (kb)**
1	51.5	44	56.9/1.63	45.05	1(F)	43.66	43.73/1.61	17.77/0.62/30.12/3.02	15.86	3,311	40.48	1.44
2	43.4	39.8	48.8/1.68	36.78	1(F)	36.14	36.21/1.62	19.86/9.91/34.77/1.57	15.15	2,456	35.69	1.47
3	47.5	40.8	52.5/0.89	37.37	1(F)	36.97	37.03/0.84	28.27/10.18/41.78/0.92	15.30	2,669	39.38	1.46
4	36.8	39	35.5/5.13	36.15	1(F)	35.80	35.85/2.61	2.65/8.79/0.98/0.84	14.55	2,368	35.38	1.39
5	33.6	33.2	34.6/1.99	30.00	1(F)	30.06	30.11/1.44	11.59/10.26/14.91/0.37	14.30	1,845	33.03	1.40
6	35.1	31.8	35.7/0.93	31.60	1	16.09	31.94/1.01	9.89/0.44/11.77/1.06	13.99	1,536	47.03	1.37
					2	15.78			14.89	1,483	44.17	1.40
7	33.1	35	32.3/1.78	30.28	1(F)	29.65	29.71/1.45	11.41/17.81/8.72/1.92	14.41	2,052	35.93	1.37
8	33.6	27.6	30.0/1.38	28.56	1(F)	28.40	28.45/1.09	18.10/2.99/5.45/0.39	13.46	2,244	39.38	1.37
9	27	21.6	26.7/6.54	30.53	1	24.50	26.53/2.77	1.77/18.58/0.64/15.08	14.16	2,112	39.19	1.48
10	23.7	26.8	23.0/3.07	23.96	1(F)	23.96	24.00/1.85	1.25/11.67/4.17/0.17	13.12	2,658	55.74	1.32
11	33.7	30.3	28.8/1.32	30.76	1	17.21	31.00/1.39	8.71/2.26/7.10/0.77	14.34	1,202	35.38	1.34
					2	12.37			14.11	931	35.55	1.39
12	30.9	30.6	25.1/1.32	27.77	1(F)	28.19	28.24/1.22	9.42/8.36/11.12/1.66	14.07	2,645	45.38	1.27
**Total**	**430**	**400.5**	**430**	**388.82**	**14**	**378.31**	**382.80/NA**	**12.33/4.62/12.33/1.57**		**29,512**		
**Ave.**									**14.41**		**40.12**	**1.39**

*Ch. = **Ch**romosome; **est. **= **est**imated; **AR **= **A**rm **R**atio; **Opt. **= **Opt**ical; **Ave. **= **Ave**rage; **Frag. **= **Frag**ment; **SD **= **S**tandard **D**eviation; **Cov. **= **Cov**erage; **F **= **F**inished; Difference = |Estimated size-Optical Map size|/Optical Map size*100%; Mol. No.: the number of single molecule maps were used to build the chromosome optical map contig; **X Cov**erage = (total mass of maps within a contig)/(chromosome optical map consensus size). ^a ^Estimated chromosome size is based on the YAC and BAC physical map [7]; ^b ^Estimated chromosome size is based on the BAC/PAC physical map [13]; ^c ^Estimated chromosome size by pachytene FISH [62]. ^d ^International Rice Genome Sequencing Project [37]. ^e^Predicted chromosome size = the optical map sizes + gap sizes [the centromere sizes in BAC and YAC map for chromosome 6 and 11] + missing small fragment sizes (Table 2).

The optical map (the fifth genome-wide physical map) of the rice genome consists of 14 optical map contigs with a total mass of 378.31 Mb – 9 are finished chromosomal contigs, and the other 5 cover the remaining 3 chromosomes (6, 9 and 11), with chromosome 6 and 11 spanned by 2 optical map contigs, harboring gaps at the centromeric regions, and with chromosome 9 spanned by 1 optical map contig, harboring a gap at the nucleolar organizer comprising ribosomal repeats (Table 1). Although, these 2 centromeric and 1 telomeric or subtelomeric gaps located within the ribosomal DNA repeat region are not confidently bridged or extended by optical mapping data, map data within these structurally important regions were used to estimate the size of such gaps, and consequently the size of these chromosomes.

### Comparison of chromosome size predicted by optical mapping and pachytene FISH

In addition to physical maps, cytogenetic data was used to size the 12 rice chromosome arm ratios using pachytene FISH [61,62]; these findings are also listed in Table 1. Compared to our optical mapping data, the size measurements for chromosome 1, 2, and 3, based on pachytene FISH data differ by more than 30%, but the size measurements for chromosome 4, 9, and 10 (< 5% difference) are very similar by these two approaches. Regarding the long and short chromosome arm ratios, it has been suggested that the long and short arms should be reversed for chromosome 3 and 6. Based on our optical mapping data, only the long and short arms for chromosome 3 need to be reversed, and the long and short arms for chromosome 6 are almost equal, with the long arm slightly longer than the short arm. The arm ratios for chromosome 4, 5, 7, 8, 9, and 10 determined by pachytene FISH (5.13, 1.99, 1.78, 1.38, 6.54 and 3.07) are quite different from those determined by optical mapping (2.61, 1.44, 1.45, 1.09, 2.82, and 1.85); however, both methods are concordant for arm ratios of 1, 2, 3, 6, 11 and 12.

### Assessment of optical mapping errors

As the rice genome sequence is finished, the accuracy of the SwaI optical maps was assessed by the comparison of optical maps against the *in silico *maps of the sequence data. As such, we used the map assembler to align *in silico *restriction maps created from both IRGSP (build 4) and TIGR (release 4) pseudomolecules against our optical mapping data. These results are shown in Table 2 and 3 which globally summarize optical mapping accuracy in terms of restriction fragment identification and sizing *vs. in silico *maps created from sequence data (pseudomolecules). The comparisons between optical maps and the *in silico *maps of the 12 pseudomolecule sequences from IRGSP showed that there were 24,504 aligned map restriction fragments with a total mass of 363.47 Mb (*in silico*) (Table 2). The comparison between optical maps and the *in silico *maps of the 12 pseudomolecule sequences from TIGR showed that there were 24,716 well-aligned map restriction fragments with a total mass of 367.14 Mb (*in silico*) (Table 3).

**Table 2:** Comparison between the optical maps and the *in silico *maps from the IRGSP pseudomolecules of rice genome sequence

					**IRGSP sequence pseudomolecule**					**Optical map**	
					
**Ch.**	**# Match frag.**	**Total match frag. mass (Mb)**	**Percent (%) out of total mass^a^**	**Ave. frag. relative sizing error(%)**	**# Telomeric gap/mass (kb)**	**Centr. gap mass (kb)**	**# Gaps Filling/Mass (kb)**	**# Gaps Calling/mass (kb)**	**# Misassembly/Mass (kb)**	**# Missing small frag./mass (kb)**	**# Missing/false cut(s)**
1	2605	42.22	96.70	3.30	2/12	1/707	5/69	6/42	3/57	87/66.38	43/4
2	2272	35.23	97.48	3.41	1/6	1/429	3/21	2/12	2/10	73/65.55	27/1
3	2312	36.75	99.40	3.37	2/37	1/438	4/496	7/75	4/239	74/63.74	45/4
4	2230	33.65	93.99	3.49	2/111	0/0	5/192	15/359	15/1036	63/51.45	36/5
5	1985	29.03	96.57	3.60	2/255	1/54	5/96	9/66	8/314	61/52.26	29/3
6	2114	30.84	96.56	3.34	2/175	1/810	2/12	3/17	1/36	80/68.71	40/5
7	1974	29.20	98.48	3.60	1/13	2/84	0/0	8/145	3/55	64/57.67	39/4
8	1999	28.00	98.59	3.37	2/16	1/24	0/0	6/104	1/39	59/53.10	30/0
9	1613	22.31	84.25	3.63	2/3503	1/320	3/51	3/16	1/55	59/52.24	42/5
10	1664	22.51	93.95	3.35	2/54	1/117	5/671	7/79	3/62	45/36.53	28/4
11	1892	27.29	88.26	3.60	2/67	1/2090	4/178	9/141	5/635	91/76.07	23/8
12	1844	26.70	94.71	3.41	2/40	1/338	0/0	9/327	7/438	59/46.55	44/2
**Total**	24,504	363.73			22/4289	12/5240	36/1786	84/1383	53/2974	815/690	426/46
**Ave.**			94.91	3.46							

*Ch. = **Ch**romosome, **Ave. **= **Ave**rage; **frag. **= **frag**ment; **Centr. **= **centr**omeric; ^a ^The predicted chromosome size based on optical map was used as the total mass to compute the percentage of the matching fragment mass. Gap or misassembly masses in the alignments were calculated based on the optical mapping data.

**Table 3:** Comparison between the optical maps and the *in silico *maps from the TIGR pseudomolecules of rice genome sequence

					**TIGR sequence pseudomolecule**					**Optical Map**	
					
**Ch.**	**# Match frag.**	**Total match frag. mass (Mb)**	**Percent (%) out of total mass^a^**	**Ave. frag. relative sizing error(%)**	**# Telomeric gap/mass (kb)**	**Centr. gap mass (kb)**	**# Gaps Filling/Mass (kb)**	**# Gaps Calling/mass (kb)**	**# Misassembly/Mass (kb)**	**# Missing small frag./mass (kb)**	**# Missing/false cut(s)**
1	2641	42.94	98.35	3.32	2/14	1/795	6/69	6/42	3/328	69/57.60	44/4
2	2301	35.52	98.28	3.45	1/10	1/466	3/17	2/12	2/14	62/52.53	33/1
3	2261	35.93	97.19	3.46	2/37	1/432	4/496	6/67	1/7.5	74/63.56	45/4
4	2279	34.63	96.73	3.57	2/112	0/0	3/65	21/621	11/435	62/51.92	36/5
5	2005	29.48	98.07	3.65	2/255	1/77	4/60	8/62	5/250	56/47.05	34/4
6	2118	30.90	96.74	3.31	2/175	1/810	1/2	3/17	1/42	73/61.90	39/4
7	1977	29.38	99.09	3.64	2/20	1/61	1/23	8/146	2/30	54/48.19	48/5
8	2009	28.10	98.94	3.49	2/23	1/24	1/1	8/245	2/68	52/45.18	37/2
9	1625	22.56	85.20	3.73	2/3451	1/302	4/54	3/20	2/57	58/52.50	42/4
10	1683	22.52	93.99	3.34	2/51	1/402	6/498	6/76	1/9	43/34.02	28/4
11	1939	28.15	91.04	3.62	2/66	1/2090	5/218	11/211	6/238	83/69.79	30/7
12	1878	27.03	95.89	3.52	2/41	1/342	0/0	11/358	3/63	58/48.18	38/2
**Total**	24,716	367.14			23/4255	11/5801	38/1503	93/1877	39/1540	744/632.39	454/48
**Ave.**			96.07	3.51							

*Ch. = **Ch**romosome, **Ave. **= **Ave**rage; **frag. **= **frag**ment; **Centr. **= **centr**omeric; ^a ^The predicted chromosome size based on optical map was used as the total mass to compute the percentage of the matching fragment mass. Gap or misassembly masses in the alignments were calculated based on the optical mapping data.

**Figure 3:**
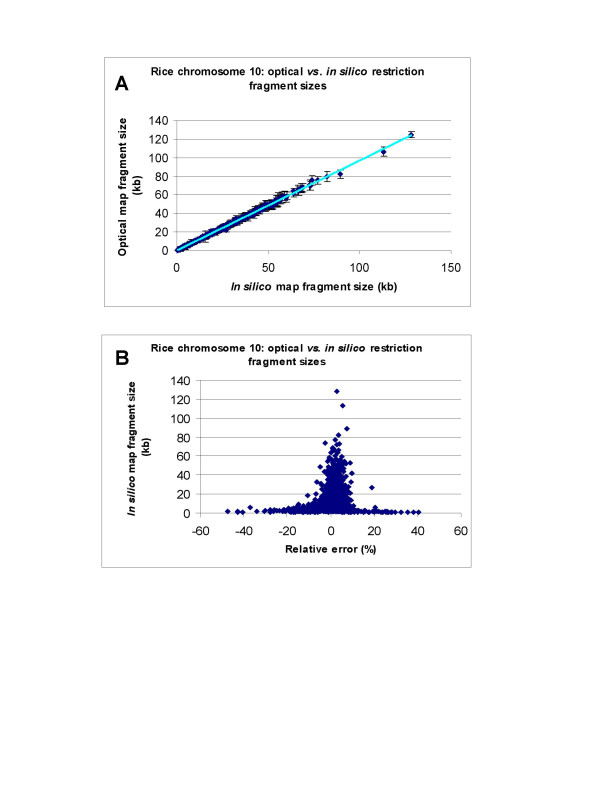
SwaI optical maps of chromosome 10 *vs. *IRGSP sequence pseudomolecule data. **A**: plot of sizing error: optical map fragments *vs. in silico *map fragment from well-aligned regions. The error bars represent the SD of optical map fragment sizes on the calculated means. **B**: plot of the relative error of optical fragment size *vs. in silico *map fragments derived from sequence data.

Fragment sizes represented by optical and corresponding *in silico *maps differ by an average of 3.46% from IRGSP (Table 2), and 3.51% from TIGR (Table 3) rice sequence data. A plot (Fig. 3A) comparing the sizes of corresponding restriction fragments within optical and *in silico *maps (IRGSP) (ch 10) show excellent agreement, confirmed by linear regression analysis (R^2 ^= 0.998), with a SD of 1.32 kb (Table 1). Such sizing accuracy was also reflected by the average absolute size difference between corresponding optical and *in silico *restriction fragments (0.34 kb for a set of 1,664 fragments of chromosome 10; average size, 12.83 kb). The total mass of the plotted optical map fragments of chromosome 10 is 22,248.57 kb, which is 264.51 kb smaller (1.18%) than the total *in silico *fragment mass from IRGSP chromosome 10 sequence.

Figure 3B is a plot of the relative error ([optical map fragment size - *in silico *map fragment size]/[*in silico *map fragment size * 100%]) for each optical map fragment against the corresponding *in silico *map fragment size from IRGSP chromosome 10 sequence. This scatter plot shows greater error for small fragments, and an average relative error of 3.35% for all map data. Among the 1,664 well-aligned restriction fragments (aligned fragments showing more than 20% relative error were excluded), 457 were less than 5 kb, and the average relative fragment sizing error compared to *in silico *map fragments was 5.60%. The average relative fragment sizing error for fragments larger than 5 kb (1,207 fragments), however, was only 2.50%. Similar results were also obtained from the comparison between chromosome 10 optical map and TIGR chromosome 10 sequence *in silico *map (data not shown). These results are consistent with previous findings [43,48,63] that concluded that the relative sizing error was inversely proportional to the fragment mass.

### Comparisons between optical maps and the rice genome sequence

**Figure 5:**
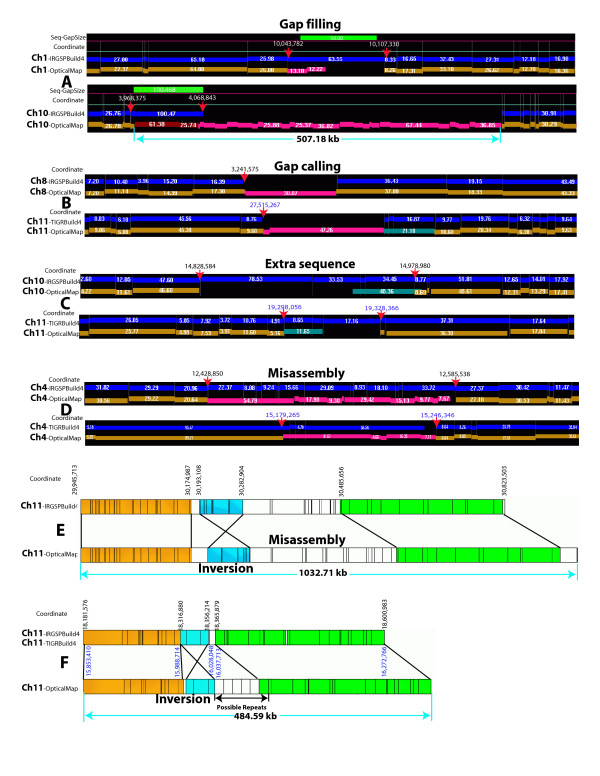
Examples of gap filing, gap calling and sequence assembly discordances detected by alignments between *in silico *(pseudomolecule sequence) and optical maps. Panels **A-D **show types of discordances revealed through alignment of optical maps with *in silico *restriction maps from IRGSPBuild4 and TIGRBuild4 pseudomolecules; red arrows show their basepair locations, and green bars highlight the size of reported gaps in pseudomolecules. Aligned *in silico *(blue) and optical maps (gold) are shown as tracks comprising individual restriction fragments drawn as numbered bars whose length scales with size (kb). Identified discordances are annotated by color-keyed bars describing restriction map features presented by optical maps, but *not *found within corresponding *in silico *restriction maps: *magenta *= consecutive restriction fragments; *red *= restriction cut site(s); *turquoise *= missing restriction site(s). **A**: The top panel (IRGSPBuild4 Ch01, 10,043,782 – 10,107,330 bp) shows an overestimated sequence gap (green bar; 50.0 kb *vs. *optical map = 13.10 kb + 12.22 kb); bottom panel (IRGSPBuild4 Ch10, 3,968,375 – 4,068,843 bp), an underestimated gap (green bar; 100.468 kb *vs. *26 optical restriction fragments = 507.18 kb, arrow). **B**: Discovered gaps in pseudomolecules: IRGSPBuild4 Ch08 (3,241,575 bp; 0.41 kb + 30.07 kb) and TIGRBuild4 Ch11 (27,515,267 bp; 2.65 kb + 47.26 kb). **C**: Extra sequence: IRGSPBuild4 Ch10 (14,828,584 – 14,978,980 bp, 150.396 kb *vs. *48.36 kb, turquoise bar); TIGRBuild4 Ch11 (19,298,056 – 19,328,366 bp, 30.310 kb *vs. *11.65 kb, turquoise bar). **D**: Misassembly: IRGSPBuild4 Ch04 (12,428,850 – 12,585,538 bp) *vs. *a stretch of 11 unaligned optical restriction fragments; TIGRBuild4 Ch04 (15,179,265 – 15,246,346 bp) *vs. *5 unaligned restriction fragments. Panels **E **and **F **show examples of large-scale misassembly of sequence. *In silico *and optical maps are horizontal tracks comprising restriction fragments demarcated by vertical lines with aligned portions color-keyed and indicated by connecting lines; unaligned restriction fragments are white. **E**: IRGSPBulid4 Ch11 (29,945,713 – 30,823,503 bp; 877.790 kb) shows an 89.796 kb inversion (blue) and two significant portions (18.121 kb, 202.752 kb; white) unaligned to the optical map. **F**: IRGSPBuild4 & TIGRBuild4 Ch11 (18,181,576 – 18,600,983 bp; 419.407 kb & 15,853,410 – 16,272,766 bp; 419.356 kb, blue lettering for TIGRBuild4) show a 39.334 kb inversion (blue), a small insertion and portion (18,356,214 – 18,365,879 bp) missing a possible repetitive region characterized by the optical map.

The above analysis of errors of the optical map *vs. *sequence on a per fragment basis is an important primary consideration for any evaluation of size measurement accuracy; however, discordances stemming from map or sequence assembly errors become apparent through global alignment procedures (map assembler, Methods) – optical *vs. *sequence alignments are shown in Fig 4. See additional data file 1 and 2 for comprehensive tables covering aforementioned discordances. The alignments between optical maps and the IRGSP sequence pseudomolecules, (Methods), identified 24,504 SwaI congruent restriction sites, 46 false cuts, 425 missing cuts, and 815 missing small fragments (mostly less than 1 kb) within the optical consensus map data. The alignments also identified 22 telomeric gaps with a total size of 4,289 kb, 12 centromeric gaps with a total estimated size of 5,240 kb, 36 sequence gaps filled by optical maps with a total size of 1,786 kb, and 82 new gaps called by optical maps within the sequence with a total size of 1,381 kb (Table 2, Fig. 4, Fig. 5A and 5B). In the IRGSP sequence pseudomolecules, sequence physical gaps were represented as consecutive "Ns", with the gap sizes defined. These gaps can be oversized or undersized through comparison to optical maps (Fig. 5A and 5B). The alignments between optical maps and the TIGR sequence pseudomolecules, (Methods), identified 24,716 SwaI congruent restriction sites, 48 false cuts, 454 missing cuts, and 744 missing small fragments (mostly less than 1 kb) within the optical consensus map data. The alignments also identified 23 telomeric gaps with a total size of 4,255 kb, 11 centromeric gaps with a total estimated size of 5,801 kb, 38 non-centromeric sequence gaps filled by optical maps with a total size of 1,503 kb, and 93 new gaps (not including centromeric and telomeric gaps) called within the sequence based on the map alignments with a total size of 1,877 kb (Table 3, Fig. 4, Fig. 5A and 5B). Within the TIGR sequence pseudomolecules, 48 physical gaps (including 10 centromeric gaps) are represented by strings of 1 kb "Ns" with gap sizes undefined. Accordingly, Figure 4 shows that larger gaps are spanned by optical maps in pseudomolecules from IRGSP as compared with those from TIGR, especially within centromeric gaps.

Some portions of the *in silico *maps of the sequence pseudomolecules could not be properly aligned with the optical maps, indicating potential sequence assembly issues – these regions are indicated in Fig. 4. Overall, there are 53 putative misassemblies, covering 2,974 kb, identified on the basis of alignments between optical and *in silico *maps of the IRGSP sequence pseudomolecules, and there are 39 putative misassemblies, covering 1,540 kb, identified on the basis of alignments between optical and *in silico *maps of the TIGR sequence pseudomolecules. These misassembly errors in the pseudomolecule data (Fig. 4, Fig. 5C, D, E, and 5F; Table 2), are categorized in three ways: 1) additional sequence in the pseudomolecule data, 2) inversions, and 3) frank large-scale misassemblies, which exhibit a series of discordances, to include gaps, or extra sequence data – typical examples are graphically depicted in Fig. 5. Some misassembly errors are shared by both IRGSP and TIGR sequence pseudomolecules such as Fig. 5F, and some may be unique to each sequence assembly such as Fig. 5C, D, and 5E. The inversion in Fig. 5F was also detected by comparing the whole-genome shotgun and map-based sequences of the rice genome.

## Discussion

We have constructed a whole genome SwaI restriction map for the rice genome (*O. sativa *ssp. *japonica *cv. Nipponbare) using a modified whole genome shotgun optical mapping approach that was used to identify problematic regions within the current sequence build. The whole genome optical map consists of 14 optical map contigs, of which 9 are finished chromosome optical map contigs. Among the remaining 3 chromosomes, chromosomes 6 and 11 each composed of two optical map contigs with gaps at the centromeric regions, and 9 only has one optical map contig with gap at the nucleolar organizer ribosomal repeat and telomeric regions. Maps, by definition, do not posses the same resolution as sequence, so that map alignments to sequence reveal discordances governed by experimental factors that hinge on the average restriction fragment size, or "resolution" of the final map, which is limited by reliable detection of small restriction fragments, sizing errors, and the extent of genome coverage [55]. Alignments between the optical and *in silico *maps, derived from IRGSP and TIGR sequence pseudomolecules or virtual contigs, enabled us to fully place such sequence data along map scaffolds. These alignments revealed a high degree of concordance (Table 2, Table 3, Fig. 3) and provided a largely independent way to assess the errors in the rice genome sequence assembly. Perhaps more importantly, map-sequence alignments have characterized a number of gaps, which may be difficult to assess using other approaches.

The estimated genome size of rice genome is 382.80 Mb – after summing the masses of all the 12 estimated chromosome sizes estimated based on optical mapping (378.31 Mb), three optical map gaps estimated based on other studies [8,64,65] (3.80 Mb), and all the missing small fragments (0.69 Mb) in the optical maps based on the map alignments between optical maps and the *in silico *maps of IRGSP sequence pseudomolecules. Comparisons with other genome or chromosome size estimations based on BAC/PAC or YAC physical maps, show that genome and chromosome size estimates based on optical mapping are mostly smaller, but were very close to the most recent estimation of genome size (388.82 Mb) based on the minimum tiles of BAC/PAC clone sequences for each chromosome [30]. However, the size estimate for chromosome 9 is quite different from that estimated by optical mapping (3.97 Mb difference, or ~15.0%). The difference between our optical map-based chromosome size and the IRGSP sequence and map-based chromosome is mainly due to the different number of copies of rice rRNA genes used for the size estimation. IRGSP used 850 copies of rice rRNA genes to estimate the length of chromosome 9 short arm nucleolar organizer DNA, which is calculated to be 6.95 Mb, but in fact, this copy number is for a diploid genome [65]. We used half of this number, which is 425 copies of rRNA genes to calculate the size of the ribosomal repeat region (3.48 Mb), because the genome sequence is haploid.

The chromosome size measurements and the arm ratios determined by pachytene FISH [61] and optical mapping are quite different. Here chromosome sizes were mostly overestimated except for chromosome 4, 9, and 10, and the arm ratios were overestimated for chromosome 4, 5, 7, 8, 9 and 10 (>10%), but were underestimated for chromosomes 6 (7.2%) and 11 (5.0%). Our results confirmed that the long and short arms of chromosome 3 should be reversed, but not for chromosome 6. The sizing discrepancies between pachytene FISH and optical mapping estimates for chromosome sizes and arm ratios reflect fundamental differences in how DNA intervals are measured and the number of markers used for analysis. In pachytene FISH, distances between markers is measured as an actual distance (microns or image pixels) using difficult to control chromatin substrates – varying degrees of condensation can affect the accuracy of distance measurements in a locus-specific way. For example, size estimate for the short arm of chromosome 4 was significantly underestimated by pachytene FISH probably due to the presence of heterochromatic or AT-rich sequences; while the sizes of chromosomes 1, 2, and 3 were significantly overestimated by pachytene FISH, because these chromosomes are mostly composed of euchromatic sequences [61,62]. These issues are largely obviated in optical mapping since the mapping substrate is just naked, fully deproteinized DNA molecules, and "distances" are robustly estimated by measurement of integrated fluorescence intensity [53,63] using a dye (YOYO-1), whose measured fluorescence intensity is somewhat insensitive to base composition or extent of DNA elongation. Consequently, the chromosome sizes presented in this paper are likely to be more accurate than those determined by pachytene FISH.

Comparisons between the optical maps and the *in silico *maps from the IRGSP and TIGR sequence pseudomolecules showed that additional gaps exist in the sequence pseudomolecules than what has been reported. There are 62 gaps (including 9 centromeric and 17 telomeric gaps) recorded in the IRGSP sequence pseudomolecules which are represented by consecutive "N" with gap sizes defined [30,31]. Sixty of the 62 gaps were bridged by optical maps, and the two gaps not bridged were located at the centromeric regions of chromosome 6 and 11. There are additional 90 gaps (including 5 telomeric and 3 centromeric gaps) plus 53 misassemblies present in the IRGSP sequence pseuodomolecules detected by the comparative analysis between optical maps and the *in silico *maps of the IRGSP sequence pseudomolecules. These misassemblies usually contain SwaI restriction site differences (Table 2, Fig. 4), and some of them may bear gaps. In TIGR rice genome sequence pseudomolecules [37], the 48 physical gaps (including 10 centromeric gaps) were recorded, and 46 of these gaps were spanned by optical maps based on the map aligments with the two unfilled gaps also located at the centromeric regions of chromosome 6 and 11. Comparative analysis between optical maps and the *in silico *maps of the TIGR sequence pseuodomolecules showed that there are additional 117 gaps (including 23 telomeric and 11 centromeric gaps), plus 39 misassemblies existed in the TIGR rice genome sequence pseudomolecules. Overall, the TIGR sequence pseudomolecules show less discordance with our findings than the IRGSP sequence pseudomolecules as judged by comparison of *in silico vs. *optical mapping fragment masses and reduced prevalence of sequence misassemblies despite discovery of additional gaps within sequence assemblies. About 70% of these gaps and 50% of of misassemblies are common to both IRGSP and TIGR sequence pseudomolecules (Fig. 4). As alignments between the optical and *in silico *maps of the sequence pseudomolecules locate gaps and their span, or possible misassembled sequences, those BAC/PAC clones from such problematic regions can be flagged for further sequence analysis. Gaps can be closed using PCR or other molecular techniques, and misassembled sequences can be corrected based on the SwaI optical restriction maps.

**Figure 6:**
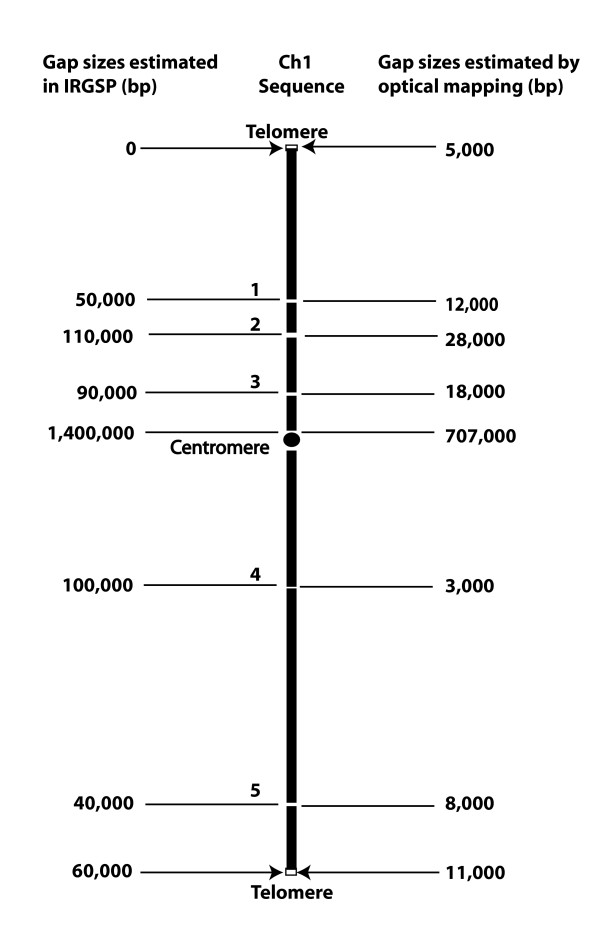
Estimates of chromosome 1 gap sizes by genetic markers or fibre (or pachytene) FISH, *vs. *optical mapping results. Diagram shows gaps (spaces between contigs), and sizes estimates among the 7 sequence contigs.

The gap sizes estimated by optical mapping are likely to be more accurate than that estimated by genetic mapping or fibre FISH and pachytene FISH physical mapping [29,33]. The genome sequence and structure of rice chromosome 1 was reported in 2002 [33] with 9 large sequence contigs and 8 gaps. Two gaps were closed and five gaps in the arm regions still remain in the current IRGSP build 4 pseudomolecules [31] The gap sizes previously estimated by genetic marker or fibre FISH and pachytene FISH are shown in Figure 6. Alignments between the *in silico *maps of the rice chromosome 1 sequence pseudomolecules and the optical maps can easily reveal the gap sizes between the contigs based on where the consecutive "Ns" located, and the gap sizes are also shown in Figure 6 based on the measurements of the optical maps. The centromeric and telomeric gaps, and the five internal chromosome arm gaps between the sequence contigs were measured to be 1,850 kb in total by genetic markers and fibre or pachytene FISH, while these gaps were measured by optical mapping were shown to be only 792 kb, which is less than one half of the estimation by genetic markers and fibre or pachytene FISH. In the TIGR sequence pseudomolecule release 4, chromosome 1 sequence pseudomolecule has six chromosome internal gaps, and five out of them are at the same locations as in IRGSP chromosome 1 sequence pseudomolecule based on the optical map (Fig. 6), however, there are 346 kb sequence inserted in gap 3 (Fig. 6), which does not belong to this region and appears to be combined from sequences of multiple chromosomes based on the optical map (data not shown). We have tried to use PCR to close the gap 4 and 5 based on IRGSP chromosome 1 sequence, and failed to generate expected unique amplicons probably due to sequence repeat elements. Other approaches are likely required in order to close these gaps.

The centromeric regions of chromosomes found in higher eukaryotes are a complex motif of repetitive sequences. Consequently, comprehensive basepair level knowledge of centromeric regions is at the frontier of genome sequencing technology presenting challenges to cloning, mapping and assembly [38,66,67]. Although rice centromeres are analyzable by optical mapping, mammalian centromeres, being larger and more complex, may challenge our ability to span regions bearing few restriction sites with ~500 kb molecules. As such, it is understandable that from previous studies that the centromeres of only chromosomes 4 and 8 have been fully mapped and sequenced. Here, we have fully mapped 10 complete centromeric regions, with the remaining chromosomes being partly characterized (Fig. 2 and Fig. 4). Accordingly, comparisons of optical map findings for the centromeric region of chromosome 8 against IRGSP and TIGR results showed that a ~24 kb gap still exists both in the IRGSP and TIGR pseudomolecules (Table 2, and Table 3), while chromosome 4 showed no discordances.

## Conclusion

A whole-genome SwaI restriction map of rice genome was constructed and comparison between the *in silico *maps of rice chromosome pseudomolecule sequences revealed not only gap characteristics, but also putatively misassembled parts of the genome sequence. We think that this whole genome optical map will greatly facilitate gap closure efforts and guide correction of misassemblies to provide an accurate and completely sequenced model for plant genome research – especially for cereal genomes. As our optical mapping system has achieved a high-degree of automation, now we can produce a whole genome optical map like rice genome with only a fraction of the cost for sequencing. We envision optical mapping employed as a new platform for comparative genomics to study closely related varieties, or cultivars, that would leverage sequence and annotation information across a broad range of rice subspecies and varieties.

## Methods

### Seed germination and DNA preparation

The seeds of rice (*O. sativa *spp. *japonica *var. Nipponbare) were washed in 10% Clorox^® ^bleach for 10 min, rinsed in sterile water (3×, ~3 min per wash), germinated on wetted brown paper towels, finally and incubated in a moist chamber at 28°C in dark for 12 days. Residual ungerminated seeds were removed from rice sprouts prior to nuclei isolation. About 5 gm of fresh sprouts were frozen in liquid nitrogen, and ground to fine powder in a pre-cooled mortar and pestle. The powder was transferred to a 50 ml conical tube, and then 30 ml of nuclei isolation buffer (NIB: 10 mM Tris-HCl, pH 9.5, 20 mM EDTA, 100 mM KCl, 0.5 M sucrose, 1.0 mM spermidine, 1.0 mM spermine, 0.15% mercaptoethanol) was added – all components were pre-chilled on ice. The powder and the buffer were mixed by slowly inverting the tube 5–10 times – the mixture was filtered through two layers of cheese cloth and two layers of Miracloth^® ^(Calbiochem, La Jolla, CA). Exactly 3 ml of NIB containing 10% (V/V) Triton X-100 were added to the filtrate and gently mixed, followed by centrifugation at 2,000 × g for 10 min at 4°C. The supernatant was removed by aspiration and the nuclei pellet was resuspended in a solution of 30 ml of NIB followed by 3 ml of NIB containing 10% Triton X-100. The suspension was centrifuged at 2,000 × g for 10 min at 4°C. The supernatant was aspirated off, and the pellet was resuspended in 1 ml NIB without mercaptoethanol but with added 30% glycerol, final concentration – 100 μl aliquots (0.5 ml Eppendorf tube) were stored at -80°C; prior to use, the nuclei were washed 2× with fresh NIB to remove glycerol. Rapid DNA concentration assays were made by lying small nuclei aliquots (TE with 1 mg/ml proteinase K with added adenovirus DNA – 25 pg/μl; internal sizing standard; Invitrogen, Carlsbad, CA), followed by mounting, restriction digestion, staining and microscope inspection. Appropriate dilutions (optimized for minimal molecule crossovers) for mapping were made by mixing isolated nuclei with 1 mg/ml proteinase K, 25 pg/μl adenovirus DNA in TE, using a wide-bore pipette tip by slowly pipetting up and down several times, followed by incubation at 65°C for 1 hr, and 37°C overnight. Such samples were mounted onto optical mapping surfaces and examined by fluorescence microscopy to assess DNA integrity, and concentration of both genomic and reference standard DNA molecules.

### Surface preparation

Surface preparation was done as previously described [48]. Briefly, glass cover slips (22 × 22 mm, Fisher's Finest) were cleaned by boiling Nano-Strip (Cyantek Corp, Freemont, CA), acidified by boiling concentrated HCl, rinsed extensively using running high purity water and ethanol with sonication, and derivatized using trimethyl and vinyl silanes to confer a positive charge and the means to crosslink the acrylamide overlay to the surface. Surfaces were evaluated by mounting and digesting lambda DASHII bacteriophage DNA with 40 units of SwaI enzyme, diluted in 100 μL of digestion buffer containing 0.02% Triton X-100, at room temperature to determine optimal digestion time, which ranged from 30 min to 2.5 hrs.

### DNA mapping

Genomic DNA molecules with added adenovirus DNA (sizing standard) were deposited as stripes on derivatized glass surfaces using a silastic microchannel system [68]. After DNA molecules were mounted, a thin layer of acrylamide (12 μL 3.3% acrylamide containing 29 parts of acrylamide, and 1 part of bis-acrylamide with 0.004% Triton X-100, 0.008% TEMED, and 0.075% ammonium persulfate) was applied to a surface: crosslinks formed between acrylamide and vinyl silane groups on the surface retain small DNA fragments and dampen fluid convection. The added detergent promotes wetting action to mediate hydrophobic patches left after peeling silastic devices from surfaces. The polymerization time of the acrylamide overlay was controlled to be ~20–30 min in a humidified chamber at room temperature to optimize restriction digestion. Mounts were washed with 400 μL TE twice for 2 min, followed by 200 μL enzyme digestion buffer for another 2 min. Then 200 μL of digestion mix was added [20 μL, NEB (New England Biolabs), buffer 2; 2 μL, 2% Triton X-100; 166 μL, deionized water; 3 μL, NEB SwaI – 20 U/μL] followed by incubation in a moist chamber at room temperature for 30 min to 2.5 hrs. After digestion, surfaces were washed 3 times with TE; gentle aspiration removed washes. To stain, surfaces were mounted on slides, pre-spotted with 12 μL of 0.2 μM YOYO-1 solution (5% YOYO-1; Molecular Probes, Eugene, OR, in TE containing 20% B-mercaptoethanol. Finally, slides were sealed with clear nail polish, and kept in the dark for 20 min or overnight at 4°C to ensure complete staining before checking samples by fluorescence microscopy.

### Image acquisition and processing

Fully automated image acquisition and processing were used to generate the map data [68]; Some single molecule maps used to span centromeric regions were manually marked-up to produce map data [44]. Briefly the imaging system consists of an argon ion laser illuminated inverted Zeiss 135 M microscope, equipped with a 63× Zeiss plan-neofluor oil immersion objective, a Dage SIT68GL low light-level video camera connected to a Sony monitor for visual inspection of the sample, and a Roper Scientific cooled charge-coupled device digital camera (Photometrics CoolSNAP_*HQ*_, 1392 × 1040 pixels, Sony ICX285 chip, 12-bit digitization) for acquiring focus and high-resolution images. A Ludl Electronics x-y stage and focus motor with 0.1 μm resolution was used for x-y-z translation. All microscope and camera functionalities are under complete computer control; the user simply aligns several fiduciary points on the surface, and the sample is imaged automatically. Consecutive images had a 20% overlap to ensure that usable data is extractable from DNA molecules spanning more than one image frame. Approximately 120 images were collected per microchannel, with 10 or 48 microchannels per surface. An entire surface (~5,000 images) can be acquired in ~4 hours due to new image acquisition software, high intensity laser illumination and a high speed CCD camera. Co-mounted adenovirus DNA molecules were used to estimate the digestion rate and to provide internal fluorescence standards for accurately sizing the DNA fragments. In total, there were 728,850 separate images presented on ~150 surfaces. Given the 6 "Genome Zephyr" imaging instruments, now functional in our laboratory, this sample load translates in to ~4 days of image acquisition, or ~25 days on a single imaging station. Newer imaging advances developed in our laboratory have further reduced the imaging time per surface to ~2.5 hours. Because our machine vision operates "in real time," images were processed as quickly as they were acquired.

### Map assembly

Whole-genome optical maps were constructed by using large, randomly sheared, single genomic DNA molecules digested to form ordered restriction maps. The map assembler and the pairwise aligner (unpublished) were used to leverage finished sequence information and assemble chromosome-wide map contigs. The map assembler uses Bayesian inference techniques and an efficient dynamic programming algorithm, which has been described previously [44,48,50,57,58].

The map aligner expects global similarity between the two maps and works well if the two maps to be compared are very similar – alignments can be made between optical maps against other optical maps, *in silico *maps derived from sequence, and consensus maps derived from contigs constructed by the map assembler from optical maps. However, global alignment approaches will fail to detect local significant instances of variation, such as insertions or deletions, which are expected when comparing optical maps derived from DNA molecules with a consensus or *in silico *map derived from sequence data. Our approach for extracting multiple high-scoring alignments is based on an efficient linear scaling approach of Huang and Miller [69]. We generate confidence scores (p-values) using an approach similar to that used by Waterman and Vingron [70] for sequence alignments. Given the large number of optical maps requiring efficient alignment with a variety of sources, we used cluster computing as described below.

### Cluster computing

A cluster computing system "Condor" [56] was used for rice genome optical map assembly. Condor is a distributed system for running computationally intensive jobs with a checkpointing function. While similar to a traditional batch queuing and scheduling system, Condor provides the additional capability of running jobs on idle desktop workstations with no special programming required to use Condor's checkpoint and remote system call features. Due to its checkpointing function, Condor is able to transparently migrate a job to a different machine when the current machine becomes actively used. Condor also allows jobs to run both locally and on multiple remote Condor sites, providing compute power on a massive scale. The Grid Laboratory of Wisconsin (GLOW) is one such Condor site that jobs can be migrated to. GLOW is an enterprise level computer grid deployed across six sites at the UW-Madison. The currently installed resources include 354 machines each with two 2.8 GHz Intel Xeon CPUs, either 2 or 4 GB of memory, 100 GB of local disk, and Gigabit ethernet.

## Authors' contributions

SZ contributed to the design of the study, carried out partial data collection, performed optical map assembly and comparison between optical map and sequence, and drafted the manuscript. MCB contributed to the data collection and manual curation of the discordance between optical map and sequence. MP contributed to data collection. CPC involved in data analysis. LP developed new protocols. SAL provided rice seed and assisted in DNA preparations. RR maintained and advanced the imaging system used for this study. SG assisted map assembly efforts and statistical analysis. DKF and ML involved in cluster computing. DCS conceived the study, contributed to experimental design and analysis, and coordinated final writing.

## Supplementary Material

Additional file 1 **Discordances between optical map and IRGSP sequence data**. This file is a table with the discordance types displayed in Fig. 4, and their chromosome positions in base pairs based on the comparison between optical map and the IRGSP genome sequence. In the note column, some information about the alignment between optical map and IRGSP sequence are provided for some of the discordance types. Additional data useful for sequence finishing is available at our website: Click here for file

Additional file 2 **Discordances between optical map and TIGR sequence data**. This file is a table with the discordance types displayed in Fig. 4, and their chromosome positions in base pairs based on the comparison between optical map and the TIGR genome sequence. In the note column, some information about the alignment between optical map and TIGR sequence are provided for some of the discordance types. Additional data useful for sequence finishing is available at our website: Click here for file
