# Supplementary material for: Validation of rice genome sequence by optical mapping
Source: BMC Genomics. 2007 Aug 15;8:278. doi: 10.1186/1471-2164-8-278 (PMC2048515; doi:10.1186/1471-2164-8-278)
Supplement: Additional file 1 — Discordances between optical map and IRGSP sequence data. This file is a table with the discordance types displayed in Fig. 4, and their chromosome positions in base pairs based on the comparison between optical map and the IRGSP genome sequence. In the note column, some information about the alignment between optical map and IRGSP sequence are provided for some of the discordance types. Additional data useful for sequence finishing is available at our website: [file 1471-2164-8-278-S1.pdf]

Additional file 1. Discordances between optical map and IRGSP sequence data**

Type of Discordances	Ch.	Start	End	Note
False_cut_on_optical_map	1	7649	7649	
False_cut_on_optical_map	1	20441464	20441464	
False_cut_on_optical_map	1	33025086	33025086	
False_cut_on_optical_map	1	29037434	29037434	
False_cut_on_optical_map	2	30044649	30044649	
False_cut_on_optical_map	3	12750657	12750657	
False_cut_on_optical_map	3	23522890	23522890	
False_cut_on_optical_map	3	33298465	33298465	
False_cut_on_optical_map	3	33826497	33826497	
False_cut_on_optical_map	4	4889434	4889434	
False_cut_on_optical_map	4	7086257	7086257	
False_cut_on_optical_map	4	27282914	27282914	
False_cut_on_optical_map	4	33183113	33183113	
False_cut_on_optical_map	4	33838933	33838933	
False_cut_on_optical_map	5	3529018	3529018	
False_cut_on_optical_map	5	9061417	9061417	
False_cut_on_optical_map	5	12240898	12240898	
False_cut_on_optical_map	5	15370306	15370306	
False_cut_on_optical_map	6	31922257	31922257	
False_cut_on_optical_map	6	17220206	17220206	
False_cut_on_optical_map	6	19317748	19317748	
False_cut_on_optical_map	6	12115279	12115279	
False_cut_on_optical_map	6	599847	599847	
False_cut_on_optical_map	7	4977698	4977698	
False_cut_on_optical_map	7	22673514	22673514	
False_cut_on_optical_map	7	23069905	23069905	
False_cut_on_optical_map	7	24515294	24515294	
False_cut_on_optical_map	9	4557176	4557176	
False_cut_on_optical_map	9	8295949	8295949	
False_cut_on_optical_map	9	9304258	9304258	
False_cut_on_optical_map	9	17874388	17874388	
False_cut_on_optical_map	9	19388949	19388949	
False_cut_on_optical_map	10	2875048	2875048	
False_cut_on_optical_map	10	6145612	6145612	
False_cut_on_optical_map	10	14105517	14105517	
False_cut_on_optical_map	10	16852166	16852166	
False_cut_on_optical_map	11	4982368	4982368	
False_cut_on_optical_map	11	10104787	10104787	
False_cut_on_optical_map	11	10128808	10128808	
False_cut_on_optical_map	11	11233356	11233356	
False_cut_on_optical_map	11	11710627	11710627	
False_cut_on_optical_map	11	21026963	21026963	
False_cut_on_optical_map	11	25079852	25079852	
False_cut_on_optical_map	11	29913977	29913977	
False_cut_on_optical_map	12	3786230	3786230	
False_cut_on_optical_map	12	18101121	18101121	
Gap_calling	1	36685440	36685440	16.21 kb insertion
Gap_calling	1	42925901	42925901	7.69 kb insertion validated
Gap_calling	1	19091351	19091351	op16.44kb_seq11.20kb
Gap_calling	1	33565707	33579930	Seq14.22kb_op18.16kb
Gap_calling	1	31506087	31533122	Seq_27.04kb_op34.30kb
Gap_calling	2	19238457	19270565	op37.65kb_seq32.11 kb
Gap_calling	2	11919828	11943528	Seq_23.70kb_op30.03kb
Gap_calling	3	14336730	14336730	3_fragments_inserted_7.17+22.79+20.15kb
Gap_calling	3	14808219	14816154	Seq7.94kb_op9.64kb**
Gap_calling	3	17596955	17645390	seq48.44kb_op56.36kb
Gap_calling	3	27180113	27191258	seq11.14kb_op15.56kb
Gap_calling	3	29608354	29623455	seq15.10kb_op18.79kb
Gap_calling	3	37139366	37176175	seq36.81kb_op40.66kb**
Gap_calling	3	36572189	36572189	3.06kb_fragment_inserted**
Gap_calling	4	3384294	3384294	7.45kb_fragment_inserted
Gap_calling	4	15637045	15637045	3.69+11.60+43.86+35.15+8.13+9.01+15.25+4.55kb_fragment_inserted
Gap_calling	4	18478261	18478261	3.36kb_fragment_inserted**
Gap_calling	4	2342468	2351402	seq8.93kb_con9.46+9.77kb
Gap_calling	4	2646375	2647883	seq1.51kb_op1.69+4.25+4.89kb

Gap_calling	4	10478305	10533480	seq55.18kb_op62.23kb
Gap_calling	4	13677812	13721222	seq43.41kb_op47.90kb
Gap_calling	4	26072053	26078916	seq6.86kb_op9.62kb
Gap_calling	4	33299872	33319223	seq19.35kb_op27.73kb
Gap_calling	4	929540	933858	seq4.32kb_op4.70+25.46+8.14+3.71+13.22+7.59+0.52+9.45+6.89+3.40+4.04kb
Gap_calling	4	4838256	4838256	4.83+2.18kb_fragment_inserted
Gap_calling	4	8737584	8737584	4.52kb_fragment_inserted
Gap_calling	4	9395101	9395101	7.08+12.17+0.29+17.55kb_fragment_inserted
Gap_calling	4	11016759	11074066	seq1.56+2.88+35.72+17.15kb_op4.11+46.66+7.77+6.89+22.63kb
Gap_calling	4	20917354	20966747	seq49.39kb_op45.52+16.11kb
Gap_calling	5	2914305	2936456	seq22.15kb_op25.21kb
Gap_calling	5	11601005	11601005	7.33kb_fragment_inserted
Gap_calling	5	13188137	13188137	8.98kb_fragment_inserted
Gap_calling	5	15375747	15375747	10.10kb_fragment_inserted
Gap_calling	5	16612766	16616318	seq3.55kb_op7.69kb
Gap_calling	5	27559457	27559457	7.05kb_fragment_inserted
Gap_calling	5	27861245	27861245	14.42kb_fragment_inserted
Gap_calling	5	29901391	29939724	seq38.33kb_op44.16kb
Gap_calling	6	8435728	8435728	5.76kb_fragment_inserted
Gap_calling	6	19305114	19321367	seq16.25kb_op10.78+3.09+8.08kb
Gap_calling	6	24890394	24922274	Seq31.88kb_op37.65kb
Gap_calling	7	3045274	3045274	possible duplication including 5 restriction fragments(in total 42.94 kb) missing in seq but present in op
Gap_calling	7	16083298	16125030	Seq41.73kb_op47.64kb
Gap_calling	7	29139150	29148566	seq9.42kb_op15.72kb
Gap_calling	7	5480556	5546997	seq66.44kb_op44.51+34.66kb
Gap_calling	7	9467901	9467901	7.45kb_fragment_inserted
Gap_calling	7	12268150	12306610	seq38.46kb_op35.76+7.12kb
Gap_calling	7	18647429	18647429	23.33+10.36+24.37kb_fragments_inserted
Gap_calling	7	28008318	28008318	7.32kb_fragment_inserted
Gap_calling	8	726519	738868	seq12.35kb_op16.81kb
Gap_calling	8	14579410	14594515	seq15.11kb_op23.88kb
Gap_calling	8	3241575	3241575	0.41+30.07kb inserted
Gap_calling	8	16332574	16332574	15.28+6.15+5.93+3.85+9.04+9.03kb inserted
Gap_calling	8	17379172	17379172	5.82kb fragment_inserted
Gap_calling	8	24694946	24694946	4.07+1.50kb_inserted
Gap_calling	9	19166999	19209916	seq1.50+41.41kb_op49.88kb
Gap_calling	9	19858814	19868417	seq9.60kb_op6.29+6.65kb
Gap_calling	9	11255290	11256432	seq1.14kb_op6.29kb
Gap_calling	10	2898200	2944438	seq46.24kb_op62.71kb
Gap_calling	10	19025141	19073074	seq47.93kb_op66.94kb
Gap_calling	10	4863257	4863257	10.63kb_fragment_inserted
Gap_calling	10	5084346	5084346	4.99kb_fragment_inserted
Gap_calling	10	19186384	19218804	seq32.42kb_op35.66kb
Gap_calling	10	5818572	5818572	16.10kb_fragment_inserted
Gap_calling	10	10393768	10393768	8.66kb_fragment_inserted
Gap_calling	11	963784	988924	Seq25.14 op20.36+6.91+13.82 gapCalling
Gap_calling	11	10167049	10224531	seq18.63+38.86 op22.30+5.37+19.82+5.65+13.63
Gap_calling	11	22151860	22151860	11.15+6.44+7.26kb fragment insertions
Gap_calling	11	28075555	28075555	6.99 kb fragment_inserted
Gap_calling	11	24093333	24108967	Seq15.63kb_op18.44kb**
Gap_calling	11	24291509	24310926	Seq19.42kb_op25.10kb
Gap_calling	11	4189081	4189081	5.58 kb_fragment_inserted
Gap_calling	11	20175534	20175534	17.95 kb_fragment_inserted
Gap_calling+Misassembly	11	18316880	18365879	seq8.61+19.85+10.88+1.15+8.52=49.01 op3.29+10.49+19.80+8.65+11.65+11.81+11.98+12.04+12.12=101.83
Gap_calling	12	8240838	8240838	6.35+7.28+5.27kb_fragment_inserted
Gap_calling	12	15759281	15771071	Seq11.79kb_op15.14kb
Gap_calling	12	15893133	15893133	8.02+8.15 kb_fragments_inserted
Gap_calling	12	20686342	20686342	missing Sequence186.58kb:17.26+13.98+9.35+30.40+17.12+78.34+1.66+7.25+18.55
Gap_calling	12	21539949	21539949	8.07 kb fragment_ inserted
Gap_calling	12	23944506	23944506	22.08 kb fragment_inserted
Gap_calling	12	16326278	16342254	op20.83 kb_seq15.98kb
Gap_calling	12	16762798	16783428	21.29+21.02+20.94kb_possible 3 copies of repeats_inserted
Gap_calling	12	6111361	6123109	op14.84_seq11.75
Gap_filling	1	10043781	10107330	seq63.55_op13.10+12.22kb gap overestimated
Gap_filling	1	11518254	11673361	seq155.11_op34.91+13.73+24.42kb gap overestimated
Gap_filling	1	15125350	15231698	seq106.35_op34.17kb gap overestimated
Gap_filling	1	27126177	27238645	seq112.47_op15.46kb gap overestimated

Gap_filling	1	42045301	42101211	seq55.91_op24.23kb gap overestimated
Gap_filling	2	5933788	6009450	Seq75.66kb_op36.15+22.32kb, gap overestimated
Gap_filling	2	19566739	19662205	seq94.96 kb_op10.41+12.30 Kb, gap overestimated
Gap_filling	2	25635840	25721619	seq 85.78_op36.72+19.59kb gap overestimated
Gap_filling	3	16891589	17027979	seq136.39kb_op30.48+12.25+9.28+2.83+4.90+37.33+3.33+37.31kb
Gap_filling	3	31859635	31978001	seq118.37kb_op36.77+8.95+6.94+13.98+4.90+11.84+22.94+12.40kb
Gap_filling	3	4812950	4902471	seq89.52kb_op34.92+16.72+22.32 gap overestimated
Gap_filling	3	13511891	14027950	seq508.58+2.56+1.55+3.37kb_op40 fragments totalling 258.35kb, gap overestimated
Gap_filling	4	3384294	3384294	7.45kb_fragment_inserted, gap underestimated
Gap_filling	4	11987580	12060534	seq4.87+8.62+8.91+6.03+5.69+4.87+6.04+2.60+25.33kb_op28.27+8.88+2.56+6.40+6.06+9.09+6.23kb
Gap_filling	4	20184503	20242252	seq1.40+13.07+16.37+11.11+6.04+2.24+7.52kb_op2.55+8.25+2.49+19.91+14.57+11.11+1.79+9.06kb
Gap_filling	4	22066096	22247421	seq5frags_totalling_181.33kb_op14frags_totalling_190.05kb
Gap_filling	4	8370251	8493801	seq123.55kb_op59.03+1.24+13.35+23.54kb gap overestimated
Gap_filling	4	9157652	9241705	seq84.05kb_op113.54kb, gap underestimated
Gap_filling	4	20819816	20876371	seq56.55kb_op62.06kb, gap underestimated
Gap_filling	4	22350316	22682110	seq331.79kb_op36.43kb, gap overestimated
Gap_filling	5	17113786	17162579	seq48.79kb_op21.19kb, gap overestimated
Gap_filling	5	7320072	7376860	seq56.01+0.77kb_op22.45+2.36+9.87+0.44kb,gap overestimated
Gap_filling	5	21545269	21644475	seq99.21kb_op13.04+1.48+49.49kb, gap overestimated
Gap_filling	5	24918959	25027358	seq108.40kb_op12.81+13.54+4.53+63.25kb, gap overestimated
Gap_filling	5	27221681	27262023	seq40.34kb_op26.33+2.19+11.87+14.88+2.11+7.33kb, gap underestimated
Gap_filling	6	23725416	23779876	seq54.46kb_op36.91+4.95+4.88kb, gap overestimated
Gap_filling	6	10547383	10659317	seq111.93kb_op43.85kb, gap overestimated
Gap_filling	9	11978855	12031822	seq52.97kb_op13.66+3.83+12.72kb, gap overestimated
Gap_filling	9	14157645	14310115	seq111.18+41.29kb_op58.78+1.84+8.89+39.22kb, gap overestimated
Gap_filling	9	20931828	21248626	seq93.42+27.28+43.45+13.57+139.08kb_op4.16+6.82+2.49+26.79+42.22+13.61+45.83+22.19kb, gap overestimated
Gap_filling	9	19858814	19868417	seq9.60kb_op6.29+6.65kb
Gap_filling	10	6824864	6913518	seq88.65kb_op9_fragments_totalling_50.58kb
Gap_filling	10	10008505	10155829	seq147.32kb_op50.98+3.26+43.84+21.45kb
Gap_filling	10	12449667	12561032	seq111.37kb_op15.59+10.34+9.29+4.48+2.18+21.09kb
Gap_filling	10	3968375	4068843	seq100.47kb_op29_fragments_totalling_507.18kb
Gap_filling	10	7462307	7557416	seq95.11kb_op35.78+8.81+9.83+7.49+3.03+7.42+31.68kb, gap underestimated
Gap_filling	11	15166134	15264194	seq74.89+23.17 op:8.46+8.15+4.96+6.82+10.34+4.35+8.66+9.34+20.39+22.33=103.08
Gap_filling	11	8910659	8997466	Seq:86.53 op:27.59+9.60+8.66+8.32+22.46=76.63, Gap overestimated
Gap_filling	11	7289804	7322407	Seq:32.60 Op:43.35, gap underestimated
Gap_filling	11	21622456	21735641	seq:113.19 op:5.00+11.28+1.16+35.12, gap overestimated
Misassembly	1	0	36306	telomere misassembly_seq1.43+34.88kb_op9.68+29.92kb
Misassembly	1	21857134	21871082	seq9.78+2.23+1.95kb_op10.88+2.98kb
Misassembly	1	31003925	31011100	two misscuts and one false cut
Misassembly	2	2414507	2423458	extra cuts and fragments in sequence
Misassembly	2	31774568	31777369	sequence is 0.45+0.88+1.47 optical_map is 2.02+0.75kb
Misassembly	3	6773564	6908765	seq0.66+61.54+12.64+6.58+2.29+2.18+49.30kb_op50.78+2.12+2.50+6.87+0.36+12.30+36.39kb
Misassembly	3	19606225	19613728	extra sequence_7.5kb
Misassembly	3	21713465	21715657	extra sequence_seq1.73+0.46+4.03+3.06+1.30+1.85+0.85_op6.54+3.23kb
Misassembly	3	37139366	37257346	telomere misassembly_seq36.81+81.17kb_op40.66+89.88kb
Misassembly	4	3331488	3347275	extra sequence_2.92+3.69+9.18kb
Misassembly	4	3361970	3366748	extra sequence_4.78kb
Misassembly	4	4635007	4642772	extra sequence_7.77kb
Misassembly	4	9577907	9605620	seq27.71kb_op24.06kb
Misassembly	4	11649186	11729152	seq59.10+0.66+20.21kb_op53.25+0.41+16.52kb
Misassembly	4	11897948	11941483	seq43.54kb_op39.02kb
Misassembly	4	12428850	12585538	probable_inversion_14_fragments_totalling_157kb
Misassembly	4	14430459	14487748	seq15.30+2.42+2.60+4.26+2.60+5.46+4.56+11.83+8.25kb_op12.63+8.83+5.45+2.40+4.43+2.58+4.90+7.60+4.77kb
Misassembly	4	15161710	15321129	seq95.47+2.70+4.70+56.56kb_op89.21+41.61+4.66+2.74+16.35+7.11kb
Misassembly	4	16748041	16813450	probable_inversion_4_fragments_totalling_65kb
Misassembly	4	19425512	19431218	seq5.71kb_op0.89+15.36kb
Misassembly	4	26046131	26058543	seq12.41kb_op9.84kb
Misassembly	4	27079649	27216366	probable_inversion_4_fragments_totalling_137kb
Misassembly	4	32888041	33012958	seq11.94+112.98kb_op23.86+101.13kb
Misassembly	4	35808771	35925326	telomere_seq0.72+54.43kb_op91.01+9.88+3.72+4.79+7.79+34.40kb
Misassembly	5	1316949	1345426	inversion_2_fragments_totalling_28kb
Misassembly	5	1934326	1987260	seq52.93kb_op47.37kb
Misassembly	5	4689388	4709887	extra sequence_seq20.50_op18.45kb
Misassembly	5	9770658	9791799	seq8.90+1.86+10.38kb_op1.85+11.94+8.41kb
Misassembly	5	12275478	12371237	seq83.04kb_op39.60+4.63+36.32kb
Misassembly	5	29098829	29125165	probable inversion_2_fragments_totalling_21kb
Misassembly	5	29964586	30039015	telomere_misassembly_seq5frags_totalling_74.42kb_op5frags_totalling_309.28kb

Misassembly	6	47968	89682	Extra sequence_seq41.71kb_op36.37kb
Misassembly	7	1353106	1386259	extra sequence_33.15kb
Misassembly	7	23445232	23464459	seq19.23kb_op16.82kb extra sequence
Misassembly	7	24434265	24439698	extra sequence_5.43kb
Misassembly	8	26752812	26790061	seq17.37+8.12+11.76kb_op8.31+12.98+17.22kb
Misassembly	9	23788435	23843361	seq13.67+35.31+5.94kb_op35.89+14.12+13.95+3.73+11.17kb
Misassembly	10	4773782	4782684	extra sequence_8.90kb
Misassembly	10	12981438	12985434	possible inversion_seq2.44+1.55kb_op1.80+2.25kb
Misassembly	10	14828584	14978980	seq78.53+33.53+3.89+34.45kb_op48.36kb
Misassembly	11	6740759	6779556	Seq2.48+13.8+11.47+7.18+3.86_op6.08+6.73+11.23+15.36kb
Misassembly	11	11111564	11129825	seq18.26kb_op16.05kb extra sequence**
Misassembly	11	11266629	11399303	seq:49.12+10.77+24.71+0.44+7.40+10.83+7.37+22.03 op:22.51+6.80+6.82+12.68+9.18+8.71+14.25+8.68+9.32+6.01+2.91+18.97
Misassembly	11	17024965	17040514	one missing cut and 2.97 kb deletion, seq:1.30+0.87+13.38 op:1.81+10.41
Misassembly	11	30174987	30485656	Misassembly_at_telomere_seq_frags_totalling_310.67kb
Misassembly	12	13784153	13802033	seq17.988kb_op15.29kb; 2.7 kb extra sequence
Misassembly	12	10313612	10318270	two missing cuts and one extra cu
Misassembly	12	15683128	15698718	seq15.59_op12.87kb
Misassembly	12	17297998	17330565	Seq:2.66+10.35+1.81+12.96+4.79;op:1.79+13.83+3.05+10.59
Misassembly	12	20951253	21079884	possible inversion plus extra sequence includec
Misassembly	12	21587567	21596438	possible inversion containec
Misassembly	12	25258350	25383803	seq:21.71+1.91+5.38+13.14+17.94+4.75+2.87+6.52+51.25 op:54.95+6.46+2.73+17.81+13.12 extra seq included and inversion
Missing_cut_on_optical_map	1	1795138	1795138	
Missing_cut_on_optical_map	1	14263736	14263736	
Missing_cut_on_optical_map	1	14662689	14662689	
Missing_cut_on_optical_map	1	14692988	14692988	
Missing_cut_on_optical_map	1	15113929	15113929	
Missing_cut_on_optical_map	1	12100950	12100950	
Missing_cut_on_optical_map	1	12212121	12212121	
Missing_cut_on_optical_map	1	12217951	12217951	
Missing_cut_on_optical_map	1	12516225	12516225	
Missing_cut_on_optical_map	1	13373306	13373306	
Missing_cut_on_optical_map	1	18694396	18694396	
Missing_cut_on_optical_map	1	19578972	19578972	
Missing_cut_on_optical_map	1	11102232	11102232	
Missing_cut_on_optical_map	1	11408362	11408362	
Missing_cut_on_optical_map	1	11471609	11471609	
Missing_cut_on_optical_map	1	4610341	4610341	
Missing_cut_on_optical_map	1	5478015	5478015	
Missing_cut_on_optical_map	1	7600298	7600298	
Missing_cut_on_optical_map	1	20530374	20530374	
Missing_cut_on_optical_map	1	21478979	21478979	
Missing_cut_on_optical_map	1	22984003	22985003	
Missing_cut_on_optical_map	1	23722415	23722415	
Missing_cut_on_optical_map	1	23980805	23980805	
Missing_cut_on_optical_map	1	23986606	23986606	
Missing_cut_on_optical_map	1	23991644	23991644	
Missing_cut_on_optical_map	1	36110677	36110677	
Missing_cut_on_optical_map	1	35478905	35478905	
Missing_cut_on_optical_map	1	12659856	12659856	
Missing_cut_on_optical_map	1	30486019	30486019	
Missing_cut_on_optical_map	1	35655472	35655472	
Missing_cut_on_optical_map	1	18836331	18836331	
Missing_cut_on_optical_map	1	19045003	19045003	
Missing_cut_on_optical_map	1	40430490	40430490	
Missing_cut_on_optical_map	1	36469088	36469088	
Missing_cut_on_optical_map	1	43886316	43886316	
Missing_cut_on_optical_map	1	12594006	12594006	
Missing_cut_on_optical_map	1	25830604	25830604	
Missing_cut_on_optical_map	1	43082523	43082523	
Missing_cut_on_optical_map	1	21010306	21010306	
Missing_cut_on_optical_map	1	21396296	21396296	
Missing_cut_on_optical_map	1	34746553	34746553	
Missing_cut_on_optical_map	1	26295403	26295403	
Missing_cut_on_optical_map	1	30354871	30354871'	
Missing_cut_on_optical_map	2	7031994	7031994	
Missing_cut_on_optical_map	2	7130628	7130628	
Missing_cut_on_optical_map	2	7882257	7882257	

Missing_cut_on_optical_map	2	8368420	8368420
Missing_cut_on_optical_map	2	9324030	9324030
Missing_cut_on_optical_map	2	9471103	9471103
Missing_cut_on_optical_map	2	9688377	9688377
Missing_cut_on_optical_map	2	9708264	9708264
Missing_cut_on_optical_map	2	9726778	9726778
Missing_cut_on_optical_map	2	12021938	12021938
Missing_cut_on_optical_map	2	16473337	16473337
Missing_cut_on_optical_map	2	19914687	19914687
Missing_cut_on_optical_map	2	24346805	24346805
Missing_cut_on_optical_map	2	24351022	24351022
Missing_cut_on_optical_map	2	25956890	25956890
Missing_cut_on_optical_map	2	30745931	30745931
Missing_cut_on_optical_map	2	31675185	31675185
Missing_cut_on_optical_map	2	36769349	36769349
Missing_cut_on_optical_map	2	32255688	32255688
Missing_cut_on_optical_map	2	632075	632075
Missing_cut_on_optical_map	2	15396418	15396418
Missing_cut_on_optical_map	2	27641121	27641121
Missing_cut_on_optical_map	2	5675023	5675023
Missing_cut_on_optical_map	2	28425745	28425745
Missing_cut_on_optical_map	2	2050591	2050591
Missing_cut_on_optical_map	2	12233638	12233638
Missing_cut_on_optical_map	2	17514120	17514120
Missing_cut_on_optical_map	3	816635	816635
Missing_cut_on_optical_map	3	2283220	2283220
Missing_cut_on_optical_map	3	3160739	3160739
Missing_cut_on_optical_map	3	4525060	4525060
Missing_cut_on_optical_map	3	4994106	4994106
Missing_cut_on_optical_map	3	5922031	5922031
Missing_cut_on_optical_map	3	6710569	6710569
Missing_cut_on_optical_map	3	11034267	11034267
Missing_cut_on_optical_map	3	11503019	11503019
Missing_cut_on_optical_map	3	13438763	13438763
Missing_cut_on_optical_map	3	13474352	13474352
Missing_cut_on_optical_map	3	13478972	13478972
Missing_cut_on_optical_map	3	14104308	14104308
Missing_cut_on_optical_map	3	14114809	14114809
Missing_cut_on_optical_map	3	14175691	14175691
Missing_cut_on_optical_map	3	14601776	14601776
Missing_cut_on_optical_map	3	14654437	14654437
Missing_cut_on_optical_map	3	15742854	15742854
Missing_cut_on_optical_map	3	15923757	15923757
Missing_cut_on_optical_map	3	16693495	16693495
Missing_cut_on_optical_map	3	16759694	16759694
Missing_cut_on_optical_map	3	18613842	18613842
Missing_cut_on_optical_map	3	19540714	19540714
Missing_cut_on_optical_map	3	19754734	19754734
Missing_cut_on_optical_map	3	21600623	21600623
Missing_cut_on_optical_map	3	21719686	21719686
Missing_cut_on_optical_map	3	21724046	21724046
Missing_cut_on_optical_map	3	22173631	22173631
Missing_cut_on_optical_map	3	23980639	23980639
Missing_cut_on_optical_map	3	24058349	24058349
Missing_cut_on_optical_map	3	25030107	25030107
Missing_cut_on_optical_map	3	25085133	25085133
Missing_cut_on_optical_map	3	25845443	25845443
Missing_cut_on_optical_map	3	25992611	25992611
Missing_cut_on_optical_map	3	26197771	26197771
Missing_cut_on_optical_map	3	28086838	28086838
Missing_cut_on_optical_map	3	30140578	30140578
Missing_cut_on_optical_map	3	30436108	30436108
Missing_cut_on_optical_map	3	30575113	30575113
Missing_cut_on_optical_map	3	33161975	33161975
Missing_cut_on_optical_map	3	33558675	33558675
Missing_cut_on_optical_map	3	34071175	34071175
Missing_cut_on_optical_map	3	35626283	35626283

Missing_cut_on_optical_map	3	36303528	36303528
Missing_cut_on_optical_map	3	36365396	36365396
Missing_cut_on_optical_map	4	1900159	1900159
Missing_cut_on_optical_map	4	2642601	2642601
Missing_cut_on_optical_map	4	2650927	2650927
Missing_cut_on_optical_map	4	3890426	3890426
Missing_cut_on_optical_map	4	4049730	4049730
Missing_cut_on_optical_map	4	4716666	4716666
Missing_cut_on_optical_map	4	5146816	5146816
Missing_cut_on_optical_map	4	7459505	7459505
Missing_cut_on_optical_map	4	7459505	7459505
Missing_cut_on_optical_map	4	8773659	8773659
Missing_cut_on_optical_map	4	11380449	11380449
Missing_cut_on_optical_map	4	11896411	11896411
Missing_cut_on_optical_map	4	13763323	13763323
Missing_cut_on_optical_map	4	13766042	13766042
Missing_cut_on_optical_map	4	13968524	13968524
Missing_cut_on_optical_map	4	16962524	16962524
Missing_cut_on_optical_map	4	16967922	16967922
Missing_cut_on_optical_map	4	16980975	16980975
Missing_cut_on_optical_map	4	17292048	17292048
Missing_cut_on_optical_map	4	18463988	18463988
Missing_cut_on_optical_map	4	19039017	19039017
Missing_cut_on_optical_map	4	21803551	21803551
Missing_cut_on_optical_map	4	23456973	23456973
Missing_cut_on_optical_map	4	25794057	25794057
Missing_cut_on_optical_map	4	28671696	28671696
Missing_cut_on_optical_map	4	29350482	29350482
Missing_cut_on_optical_map	4	31161122	31161122
Missing_cut_on_optical_map	4	33622951	33622951
Missing_cut_on_optical_map	4	3994429	3994429
Missing_cut_on_optical_map	4	2190829	2190829
Missing_cut_on_optical_map	4	4474286	4474286
Missing_cut_on_optical_map	4	23160920	23160920
Missing_cut_on_optical_map	4	1975639	1975639
Missing_cut_on_optical_map	4	31515243	31515243
Missing_cut_on_optical_map	4	21803551	21803551
Missing_cut_on_optical_map	4	9012544	9012544
Missing_cut_on_optical_map	5	5340800	5340800
Missing_cut_on_optical_map	5	5352987	5352987
Missing_cut_on_optical_map	5	5682846	5682846
Missing_cut_on_optical_map	5	9561473	9561473
Missing_cut_on_optical_map	5	9955384	9955384
Missing_cut_on_optical_map	5	10622755	10622755
Missing_cut_on_optical_map	5	11969366	11969366
Missing_cut_on_optical_map	5	12162695	12162695
Missing_cut_on_optical_map	5	14955783	14955783
Missing_cut_on_optical_map	5	15434832	15434832
Missing_cut_on_optical_map	5	15438725	15438725
Missing_cut_on_optical_map	5	15879067	15879067
Missing_cut_on_optical_map	5	18422786	18422786
Missing_cut_on_optical_map	5	18526355	18526355
Missing_cut_on_optical_map	5	18528716	18528716
Missing_cut_on_optical_map	5	18654146	18654146
Missing_cut_on_optical_map	5	18756516	18756516
Missing_cut_on_optical_map	5	19076489	19076489
Missing_cut_on_optical_map	5	19647595	19647595
Missing_cut_on_optical_map	5	19827610	19827610
Missing_cut_on_optical_map	5	20057845	20057845
Missing_cut_on_optical_map	5	23278422	23278422
Missing_cut_on_optical_map	5	23893733	23893733
Missing_cut_on_optical_map	5	24391364	24391364
Missing_cut_on_optical_map	5	25434201	25434201
Missing_cut_on_optical_map	5	25439381	25439381
Missing_cut_on_optical_map	5	28032923	28032923
Missing_cut_on_optical_map	5	28986297	28986297
Missing_cut_on_optical_map	5	29059252	29059252

Missing_cut_on_optical_map	6	19090063	19090063
Missing_cut_on_optical_map	6	9055006	9055006
Missing_cut_on_optical_map	6	8474669	8474669
Missing_cut_on_optical_map	6	11706520	11706520
Missing_cut_on_optical_map	6	11706520	11706520
Missing_cut_on_optical_map	6	5153502	5153502
Missing_cut_on_optical_map	6	5153502	5153502
Missing_cut_on_optical_map	6	29105295	29105295
Missing_cut_on_optical_map	6	29105295	29105295
Missing_cut_on_optical_map	6	25939099	25939099
Missing_cut_on_optical_map	6	19485587	19485587
Missing_cut_on_optical_map	6	19485587	19485587
Missing_cut_on_optical_map	6	8864853	8864853
Missing_cut_on_optical_map	6	8502271	8502271
Missing_cut_on_optical_map	6	15266215	15266215
Missing_cut_on_optical_map	6	21124697	21124697
Missing_cut_on_optical_map	6	4445741	4445741
Missing_cut_on_optical_map	6	3750043	3750043
Missing_cut_on_optical_map	6	17359105	17359105
Missing_cut_on_optical_map	6	1939720	1939720
Missing_cut_on_optical_map	6	12166528	12166528
Missing_cut_on_optical_map	6	28760847	28760847
Missing_cut_on_optical_map	6	3524478	3524478
Missing_cut_on_optical_map	6	14137792	14137792
Missing_cut_on_optical_map	6	30569941	30569941
Missing_cut_on_optical_map	6	20117724	20117724
Missing_cut_on_optical_map	6	1230828	1230828
Missing_cut_on_optical_map	6	1230828	1230828
Missing_cut_on_optical_map	6	20579570	20579570
Missing_cut_on_optical_map	6	20579570	20579570
Missing_cut_on_optical_map	6	3481522	3481522
Missing_cut_on_optical_map	6	23612029	23612029
Missing_cut_on_optical_map	6	9386676	9386676
Missing_cut_on_optical_map	6	21755788	21755788
Missing_cut_on_optical_map	6	6052531	6052531
Missing_cut_on_optical_map	6	12757209	12757209
Missing_cut_on_optical_map	6	8401656	8401656
Missing_cut_on_optical_map	6	7350587	7350587
Missing_cut_on_optical_map	6	7350587	7350587
Missing_cut_on_optical_map	6	24503116	24503116
Missing_cut_on_optical_map	7	414225	414225
Missing_cut_on_optical_map	7	1902319	1902319
Missing_cut_on_optical_map	7	3977219	3977219
Missing_cut_on_optical_map	7	4282966	4282966
Missing_cut_on_optical_map	7	4847264	4847264
Missing_cut_on_optical_map	7	6002906	6002906
Missing_cut_on_optical_map	7	6619013	6619013
Missing_cut_on_optical_map	7	6643285	6643285
Missing_cut_on_optical_map	7	8557100	8557100
Missing_cut_on_optical_map	7	8586991	8586991
Missing_cut_on_optical_map	7	9615993	9615993
Missing_cut_on_optical_map	7	10114469	10114469
Missing_cut_on_optical_map	7	10119542	10119542
Missing_cut_on_optical_map	7	10528898	10528898
Missing_cut_on_optical_map	7	10535395	10535395
Missing_cut_on_optical_map	7	10944143	10944143
Missing_cut_on_optical_map	7	11336815	11336815
Missing_cut_on_optical_map	7	13656992	13656992
Missing_cut_on_optical_map	7	13934002	13934002
Missing_cut_on_optical_map	7	14768998	14768998
Missing_cut_on_optical_map	7	14840585	14840585
Missing_cut_on_optical_map	7	14971603	14971603
Missing_cut_on_optical_map	7	15650402	15650402
Missing_cut_on_optical_map	7	16078051	16078051
Missing_cut_on_optical_map	7	16080270	16080270
Missing_cut_on_optical_map	7	16165364	16165364
Missing_cut_on_optical_map	7	16615789	16615789

Missing_cut_on_optical_map	7	17880657	17880657
Missing_cut_on_optical_map	7	19637362	19637362
Missing_cut_on_optical_map	7	19931945	19931945
Missing_cut_on_optical_map	7	20497682	20497682
Missing_cut_on_optical_map	7	21245230	21245230
Missing_cut_on_optical_map	7	25134334	25134334
Missing_cut_on_optical_map	7	26445459	26445459
Missing_cut_on_optical_map	7	26858067	26858067
Missing_cut_on_optical_map	7	26860978	26860978
Missing_cut_on_optical_map	7	28856902	28856902
Missing_cut_on_optical_map	7	29727374	29727374
Missing_cut_on_optical_map	7	29611788	29611788
Missing_cut_on_optical_map	8	826169	826169
Missing_cut_on_optical_map	8	1675187	1675187
Missing_cut_on_optical_map	8	2614374	2614374
Missing_cut_on_optical_map	8	4973263	4973263
Missing_cut_on_optical_map	8	6588831	6588831
Missing_cut_on_optical_map	8	6673372	6673372
Missing_cut_on_optical_map	8	6879027	6879027
Missing_cut_on_optical_map	8	6887005	6887005
Missing_cut_on_optical_map	8	6890830	6890830
Missing_cut_on_optical_map	8	6954382	6954382
Missing_cut_on_optical_map	8	6958687	6958687
Missing_cut_on_optical_map	8	7943975	7943975
Missing_cut_on_optical_map	8	8525093	8525093
Missing_cut_on_optical_map	8	8807537	8807537
Missing_cut_on_optical_map	8	9521267	9521267
Missing_cut_on_optical_map	8	10640164	10640164
Missing_cut_on_optical_map	8	11009505	11009505
Missing_cut_on_optical_map	8	11112823	11112823
Missing_cut_on_optical_map	8	12520656	12520656
Missing_cut_on_optical_map	8	12528229	12528229
Missing_cut_on_optical_map	8	12632830	12632830
Missing_cut_on_optical_map	8	13919018	13919018
Missing_cut_on_optical_map	8	15269989	15269989
Missing_cut_on_optical_map	8	16119745	16119745
Missing_cut_on_optical_map	8	16122450	16122450
Missing_cut_on_optical_map	8	16977146	16977146
Missing_cut_on_optical_map	8	20837326	20837326
Missing_cut_on_optical_map	8	26533573	26533573
Missing_cut_on_optical_map	8	27314192	27314192
Missing_cut_on_optical_map	8	15033058	15033058
Missing_cut_on_optical_map	9	5115	5115
Missing_cut_on_optical_map	9	1053474	1053474
Missing_cut_on_optical_map	9	3538430	3538430
Missing_cut_on_optical_map	9	3738763	3738763
Missing_cut_on_optical_map	9	4393024	4393024
Missing_cut_on_optical_map	9	4637515	4637515
Missing_cut_on_optical_map	9	4751812	4751812
Missing_cut_on_optical_map	9	5638257	5638257
Missing_cut_on_optical_map	9	5694928	5694928
Missing_cut_on_optical_map	9	6306923	6306923
Missing_cut_on_optical_map	9	6313794	6313794
Missing_cut_on_optical_map	9	7496841	7496841
Missing_cut_on_optical_map	9	7623037	7623037
Missing_cut_on_optical_map	9	7917031	7917031
Missing_cut_on_optical_map	9	8107499	8107499
Missing_cut_on_optical_map	9	8200465	8200465
Missing_cut_on_optical_map	9	8330998	8330998
Missing_cut_on_optical_map	9	8712470	8712470
Missing_cut_on_optical_map	9	8778749	8778749
Missing_cut_on_optical_map	9	8835356	8835356
Missing_cut_on_optical_map	9	8887673	8887673
Missing_cut_on_optical_map	9	8892651	8892651
Missing_cut_on_optical_map	9	10267115	10267115
Missing_cut_on_optical_map	9	10408511	10408511
Missing_cut_on_optical_map	9	10416059	10416059

Missing_cut_on_optical_map	9	10649330	10649330
Missing_cut_on_optical_map	9	12192336	12192336
Missing_cut_on_optical_map	9	12717800	12717800
Missing_cut_on_optical_map	9	13309321	13309321
Missing_cut_on_optical_map	9	13509797	13509797
Missing_cut_on_optical_map	9	13532299	13532299
Missing_cut_on_optical_map	9	13675539	13675539
Missing_cut_on_optical_map	9	14968527	14968527
Missing_cut_on_optical_map	9	16660599	16660599
Missing_cut_on_optical_map	9	17871535	17871535
Missing_cut_on_optical_map	9	18378630	18378630
Missing_cut_on_optical_map	9	19275392	19275392
Missing_cut_on_optical_map	9	20878118	20878118
Missing_cut_on_optical_map	9	22018667	22018667
Missing_cut_on_optical_map	9	22245349	22245349
Missing_cut_on_optical_map	9	3946792	3946792
Missing_cut_on_optical_map	9	10114682	10114682
Missing_cut_on_optical_map	10	419184	419184
Missing_cut_on_optical_map	10	421891	421891
Missing_cut_on_optical_map	10	2101089	2101089
Missing_cut_on_optical_map	10	2213839	2213839
Missing_cut_on_optical_map	10	2578545	2578545
Missing_cut_on_optical_map	10	3688455	3688455
Missing_cut_on_optical_map	10	4562243	4562243
Missing_cut_on_optical_map	10	4860300	4860300
Missing_cut_on_optical_map	10	5098361	5098361
Missing_cut_on_optical_map	10	5104733	5104733
Missing_cut_on_optical_map	10	5181474	5181474
Missing_cut_on_optical_map	10	5833294	5833294
Missing_cut_on_optical_map	10	7690027	7690027
Missing_cut_on_optical_map	10	9376210	9376210
Missing_cut_on_optical_map	10	9394865	9394865
Missing_cut_on_optical_map	10	10711039	10711039
Missing_cut_on_optical_map	10	10717891	10717891
Missing_cut_on_optical_map	10	10782460	10782460
Missing_cut_on_optical_map	10	11359671	11359671
Missing_cut_on_optical_map	10	11866465	11866465
Missing_cut_on_optical_map	10	12778655	12778655
Missing_cut_on_optical_map	10	13409642	13409642
Missing_cut_on_optical_map	10	13654534	13654534
Missing_cut_on_optical_map	10	13943432	13943432
Missing_cut_on_optical_map	10	16425284	16425284
Missing_cut_on_optical_map	10	16927706	16927706
Missing_cut_on_optical_map	10	19405269	19405269
Missing_cut_on_optical_map	10	23194644	23194644
Missing_cut_on_optical_map	11	9540166	9540166
Missing_cut_on_optical_map	11	10135448	10135448
Missing_cut_on_optical_map	11	10613813	10613813
Missing_cut_on_optical_map	11	14689570	14689570
Missing_cut_on_optical_map	11	16921995	16921995
Missing_cut_on_optical_map	11	17884366	17884366
Missing_cut_on_optical_map	11	17919205	17919205
Missing_cut_on_optical_map	11	18259547	18259547
Missing_cut_on_optical_map	11	18383210	18383210
Missing_cut_on_optical_map	11	21030931	21030931
Missing_cut_on_optical_map	11	19728222	19728222
Missing_cut_on_optical_map	11	19867538	19867538
Missing_cut_on_optical_map	11	20785105	20785105
Missing_cut_on_optical_map	11	21323963	21323963
Missing_cut_on_optical_map	11	22164486	22164486
Missing_cut_on_optical_map	11	24772301	24772301
Missing_cut_on_optical_map	11	26382239	26382239
Missing_cut_on_optical_map	11	27303290	27303290
Missing_cut_on_optical_map	11	30104468	30104468
Missing_cut_on_optical_map	11	1949958	1949958
Missing_cut_on_optical_map	11	3802373	3802373
Missing_cut_on_optical_map	11	5704982	5704982

Missing_cut_on_optical_map	11	5868622	5868622
Missing_cut_on_optical_map	12	1440482	1440482
Missing_cut_on_optical_map	12	1866893	1866893
Missing_cut_on_optical_map	12	2789305	2789305
Missing_cut_on_optical_map	12	18870683	18870683
Missing_cut_on_optical_map	12	18874645	18874645
Missing_cut_on_optical_map	12	19132114	19132114
Missing_cut_on_optical_map	12	19365328	19365328
Missing_cut_on_optical_map	12	19599397	19599397
Missing_cut_on_optical_map	12	20023244	20023244
Missing_cut_on_optical_map	12	20384864	20384864
Missing_cut_on_optical_map	12	20384864	20384864
Missing_cut_on_optical_map	12	20506327	20506327
Missing_cut_on_optical_map	12	20992420	20922420
Missing_cut_on_optical_map	12	21333290	21333290
Missing_cut_on_optical_map	12	21537847	21537847
Missing_cut_on_optical_map	12	21579646	21579646
Missing_cut_on_optical_map	12	21890257	21890257
Missing_cut_on_optical_map	12	22481041	22481041
Missing_cut_on_optical_map	12	23856603	23856603
Missing_cut_on_optical_map	12	27168420	27168420
Missing_cut_on_optical_map	12	27171551	27171551
Missing_cut_on_optical_map	12	20023244	20023244
Missing_cut_on_optical_map	12	3603181	3603181
Missing_cut_on_optical_map	12	3698348	3698348
Missing_cut_on_optical_map	12	4821656	4821656
Missing_cut_on_optical_map	12	4824121	4824121
Missing_cut_on_optical_map	12	5501843	5501843
Missing_cut_on_optical_map	12	6251130	6251130
Missing_cut_on_optical_map	12	7591337	7591337
Missing_cut_on_optical_map	12	14465629	14465629
Missing_cut_on_optical_map	12	14524676	14524676
Missing_cut_on_optical_map	12	16232667	16232667
Missing_cut_on_optical_map	12	16234802	16234802
Missing_cut_on_optical_map	12	8196732	8196732
Missing_cut_on_optical_map	12	8500833	8500833
Missing_cut_on_optical_map	12	8677851	8677851
Missing_cut_on_optical_map	12	10592005	10592005
Missing_cut_on_optical_map	12	11443593	11443593
Missing_cut_on_optical_map	12	12811678	12811678
Missing_cut_on_optical_map	12	12876436	12876436
Missing_cut_on_optical_map	12	12902080	12902080
Missing_cut_on_optical_map	12	12946285	12946285
Missing_cut_on_optical_map	12	12957370	12957370
Missing_cut_on_optical_map	12	13440898	13440898

*Start=start position based sequence; End=end position based on sequence; seq=sequence; op=optical map; Ch=chromosome;**with less confidence
