# Supplementary material for: Validation of rice genome sequence by optical mapping
Source: BMC Genomics. 2007 Aug 15;8:278. doi: 10.1186/1471-2164-8-278 (PMC2048515; doi:10.1186/1471-2164-8-278)
Supplement: Additional file 2 — Discordances between optical map and TIGR sequence data. This file is a table with the discordance types displayed in Fig. 4, and their chromosome positions in base pairs based on the comparison between optical map and the TIGR genome sequence. In the note column, some information about the alignment between optical map and TIGR sequence are provided for some of the discordance types. Additional data useful for sequence finishing is available at our website: [file 1471-2164-8-278-S2.pdf]

Additional file 2. Discordances between optical map and TIGR rice sequence data*

Type od Discordance	Ch	Start	End	Note
False_cut_on_optical_map	1	19111466	19111466	
False_cut_on_optical_map	1	27084673	27084673	
False_cut_on_optical_map	1	27609399	27609399	
False_cut_on_optical_map	1	31596088	31596088	
False_cut_on_optical_map	2	33949207	33949207	
False_cut_on_optical_map	3	12677491	12677491	
False_cut_on_optical_map	3	22717316	22717316	
False_cut_on_optical_map	3	32386706	32386706	
False_cut_on_optical_map	3	32914737	32914737	
False_cut_on_optical_map	4	4877306	4877306	
False_cut_on_optical_map	4	7073707	7073707	
False_cut_on_optical_map	4	26665280	26665280	
False_cut_on_optical_map	4	32565511	32565511	
False_cut_on_optical_map	4	33221331	33221331	
False_cut_on_optical_map	4	11577636	11636731	
False_cut_on_optical_map	4	11637395	11656270	
False_cut_on_optical_map	5	3528990	3528990	
False_cut_on_optical_map	5	9029659	9029659	
False_cut_on_optical_map	5	12208557	12208557	
False_cut_on_optical_map	5	15314408	15314408	
False_cut_on_optical_map	6	599834	599834	
False_cut_on_optical_map	6	12046270	12046270	
False_cut_on_optical_map	6	16341917	16341917	
False_cut_on_optical_map	6	31044686	31044686	
False_cut_on_optical_map	7	4943846	4943846	
False_cut_on_optical_map	7	22011663	22011663	
False_cut_on_optical_map	7	22408406	22408406	
False_cut_on_optical_map	7	22733022	22733022	
False_cut_on_optical_map	7	23853442	23853442	
False_cut_on_optical_map	8	16419977	16419977	
False_cut_on_optical_map	8	24331818	24331818	
False_cut_on_optical_map	9	3990463	3990463	
False_cut_on_optical_map	9	7729237	7729237	
False_cut_on_optical_map	9	18734237	18734237	
False_cut_on_optical_map	9	17219676	17219676	
False_cut_on_optical_map	10	2880186	2880186	
False_cut_on_optical_map	10	13416687	13416687	
False_cut_on_optical_map	10	6230172	6230172	
False_cut_on_optical_map	10	16065563	16065563	
False_cut_on_optical_map	11	4994744	4994744	
False_cut_on_optical_map	11	8193737	8193737	
False_cut_on_optical_map	11	10038149	10038149	
False_cut_on_optical_map	11	10062170	10062170	
False_cut_on_optical_map	11	11166430	11166430	
False_cut_on_optical_map	11	18698098	18698098	
False_cut_on_optical_map	11	22736425	22736425	
False_cut_on_optical_map	12	17927826	17927826	
False_cut_on_optical_map	12	3786177	3786177	
Gap_calling	1	16887306	16900557	seq13.25kb_op15.17kb**
Gap_calling	1	17761353	17773092	seq11.20+0.54kb_op16.44kb
Gap_calling	1	30077089	30104124	seq27.04kb_op34.30kb
Gap_calling	1	32136709	32150932	seq14.22kb_op18.16kb
Gap_calling	1	35256442	35256442	16.21kb_fragment_inserted
Gap_calling	1	41457903	41457903	7.69kb_fragment_inserted
Gap_calling	2	18453727	18485835	seq32.11kb_op37.65kb
Gap_calling	2	11890828	11914528	seq23.70kb_op30.03kb
Gap_calling	3	13764564	13764564	7.17+22.79+20.15kb_fragment_inserted
Gap_calling	3	16915988	16964423	seq48.44kb_op56.36kbgap7.92kb
Gap_calling	3	26368898	26368898	seq11.14kb_op15.56kb_gap4.42kb
Gap_calling	3	35660390	35673615	seq13.22kb_op3.06+14.88kb_gap4.72kb
Gap_calling	3	28797099	28812200	seq15.10kb_op18.79kb_gap3.69kb
Gap_calling	3	26213651	26233376	seq19.73kb_op23.87kb_gap4.14kb
Gap_calling	4	9086102	9170155	seq84.05kb_op113.54kb_gap29.49kb
Gap_calling	4	10406755	10461930	seq55.18kb_op62.23kb_gap7.05kb
Gap_calling	4	13602975	13646385	seq43.41kb_op47.90kb_gap4.49kb

Gap_calling	4	19987680	20004894	seq17.21kb_op19.91kb_gap2.70kb
Gap_calling	4	20052428	20066662	seq13.36+0.87kb_op19.19_gap4.96 kb
Gap_calling	4	20610119	20666674	seq56.55kb_op62.06kb_gap5.51kb
Gap_calling	4	20707657	20757050	seq49.39kb_op45.52+16.11kb_gap12.24kb
Gap_calling	4	25454419	25461282	seq6.86kb_op9.62kb_gap2.76kb**
Gap_calling	4	32682270	32701621	seq19.35kb_op27.73kb_gap8.38kb
Gap_calling	4	929500	929500	seq4.32+23.79kb_op4.70+25.46+8.14+3.71+13.22+7.59+0.52+9.45+6.89+3.40kb_gap54.97kb
Gap_calling	4	2351362	2351362	9.77kb_fragment_inserted
Gap_calling	4	2647843	2647843	4.25+4.89kb_fragments_inserted
Gap_calling	4	4825886	4825886	4.83+2.18kb_fragments_inserted
Gap_calling	4	8666034	8666034	4.52kb_fragment_inserted
Gap_calling	4	9323551	9323551	7.08+12.17+17.55kb_fragments_inserted
Gap_calling	4	10949645	11002516	seq35.72+17.15kb_op46.67+7.77+6.89+22.63kb31.09kb
Gap_calling	4	15532794	15532794	tandem_repeat_inserted_twice
Gap_calling	4	18269895	18269895	3.36kb_fragment_inserted
Gap_calling	4	19217146	19222852	seq5.71kb_op0.89+15.36_gap10.54kb
Gap_calling	4	20031039	20037766	seq6.73kb_op1.79+9.06kb_gap4.12kb
Gap_calling	4	21916561	21928819	seq12.26kb_op27.53+11.33+5.60+16.80+7.76+28.77+2.53+6.34kb111.2kb
Gap_calling	5	2914277	2936428	seq22.15kb_op25.21kb3.06kb
Gap_calling	5	16556808	16596307	seq39.50kb_op7.69+37.18kb5.37kb
Gap_calling	5	11569247	11569247	7.33kb_fragment_inserted
Gap_calling	5	13132619	13132619	8.98kb_fragment_inserted
Gap_calling	5	15319849	15319849	10.10kb_fragment_inserted
Gap_calling	5	27394605	27394605	7.05kb_fragment_inserted
Gap_calling	5	27696393	27696393	14.42kb_fragment_inserted
Gap_calling	5	29736539	29774872	seq38.33kb_op44.16kb5.83kb
Gap_calling	6	18427114	18443367	seq16.25kb_op10.78+3.09+8.08kb
Gap_calling	6	8435728	8435728	5.76kb_fragment_inserted
Gap_calling	6	24012394	24044274	seq31.88kb_op37.65kb
Gap_calling	7	3091860	3091860	9.32+3.09+25.16+1.29+5.11kb_fragments_inserted
Gap_calling	7	5446704	5513145	seq66.44kb_op44.51+34.66kb
Gap_calling	7	9434049	9434049	7.45kb_fragment_inserted
Gap_calling	7	11905298	11943758	seq38.46kb_op35.76+7.12kb
Gap_calling	7	15421446	15463178	seq41.73kb_op47.64kb
Gap_calling	7	17985577	17985577	23.33+10.36+24.37kb_fragments_inserted
Gap_calling	7	27346466	27346466	7.32kb_fragment_inserted
Gap_calling	7	28477298	28486714	seq9.42kb_op15.72kb
Gap_calling	8	12494258	12512179	seq11.30+6.62kb_op4.00+9.18+5.50kb
Gap_calling	8	12907443	12974754	seq67.31kb_op104.96kb
Gap_calling	8	3236228	3236228	0.41+30.07kb_fragments_inserted
Gap_calling	8	13074484	13074484	57.23+46.01kb_fragments_inserted
Gap_calling	8	16111726	16111726	15.28+6.15+5.93+3.85+9.04+9.03kb_fragments_inserted
Gap_calling	8	17158324	17158324	5.82kb_fragment_inserted
Gap_calling	8	24474098	24474098	4.07+1.50kb_fragments_inserted
Gap_calling	8	721172	733521	seq12.35kb_op16.81kb
Gap_calling	8	14358562	14373667	seq15.11kb_op23.88kb
Gap_calling	9	10688578	10689720	seq1.14kb_op6.29kb
Gap_calling	9	13551933	13655403	seq62.18+41.29kb_op58.78+1.84+8.89+39.22kb
Gap_calling	9	14269944	14290928	seq20.98kb_op26.82kb
Gap_calling	9	18512287	18555204	seq1.50+41.41kb_op49.88kb
Gap_calling	9	18708089	18759105	seq51.02kb_op28.18+26.90kb
Gap_calling	9	19204691	19214294	seq9.60kb_op6.29+6.65kb
Gap_calling	10	2903339	2949577	seq46.24kb_op62.71kb
Gap_calling	10	18238334	18286267	seq47.93kb_op66.94kb
Gap_calling	10	4948736	4948736	10.63kb_fragment_inserted
Gap_calling	10	5169825	5169825	4.99kb_fragment_inserted
Gap_calling	10	5904051	5904051	16.10kb_fragment_inserted
Gap_calling	10	9805325	9805325	8.66kb_fragment_inserted
Gap_calling	11	4201773	4242234	seq40.46kb_op5.77+42.03kb
Gap_calling	11	10100411	10157893	seq18.63+38.86kb_op22.30+5.55+20.49+5.84+14.09kb
Gap_calling	11	15261000	15277256	seq16.26kb_op18.77kb
Gap_calling	11	15988714	16037713	seq8.61+19.85+10.88+1.15+8.52kb_op3.40+10.84+20.46+8.94+12.04+12.20+12.38+12.44+12.52kb
Gap_calling	11	21947646	21967063	seq19.42kb_op25.10kb
Gap_calling	11	28383682	28439525	seq55.84kb_op62.15kb
Gap_calling	11	17846350	17846350	18.55kb_fragment_inserted
Gap_calling	11	21749470	21765104	seq15.63kb_op18.44+1.36kb
Gap_calling	11	25417296	25417296	5.85kb_fragment_inserted

Gap_calling	11	25726068	25726068	7.22kb_fragment_inserted
Gap_calling	11	28017212	28017212	33.63+6.35+2.05+34.65+6.33+3.65kb_fragments_inserted
Gap_calling	12	6111200	6122948	seq11.75kb_op14.84kb
Gap_calling	12	8239188	8239188	6.35+7.28+5.27kb_fragments_inserted
Gap_calling	12	15608510	15620300	seq11.79kb_op15.14kb
Gap_calling	12	15742362	15742362	8.02kb_fragment_inserted
Gap_calling	12	16175507	16191483	seq15.98kb_op20.83kb
Gap_calling	12	16612024	16612024	21.29+21.02+20.94kb_fragments_inserted
Gap_calling	12	17147224	17179791	seq2.66+10.35+1.81+12.96+4.79kb_op1.79+13.83+3.05+10.59kb
Gap_calling	12	17735482	17735482	21.90+0.61kb_fragments_inserted
Gap_calling	12	20513019	20513019	17.26+13.98+9.35+30.40+17.12+78.34+1.66+7.25+18.55kb_fragments_inserted
Gap_calling	12	21322149	21322149	8.07kb_fragment_inserted
Gap_calling	12	21369767	21369767	9.45kb_fragment_inserted
Gap_calling	12	23707251	23707251	22.08kb_fragment_inserted
Gap_filling	1	10056152	10056152	12.22kb_fragment_inserted
Gap_filling	1	15297549	15326905	seq29.36kb_op34.17kb gap underestimated
Gap_filling	1	11467076	11513183	seq46.11kb_op34.91+13.73+24.42kb gap underestimated
Gap_filling	1	25796179	25809647	seq13.47kb_op15.46kb gap underestimated
Gap_filling	1	40616303	40633213	seq16.91kb_op24.23kb gap underestimated
Gap_filling	1	14973255	14974255	seq8.00kb-op17.00kb gap underestimated
Gap_filling	1	15317640	15318640	seq8.00kb-op17.00kb gap underestimated
Gap_filling	2	5933788	5980450	seq46.66kb_op36.15+22.32kb gap underestimated
Gap_filling	2	18782009	18798475	seq0.51+15.96kb_op10.41+12.30kb gap underestimated
Gap_filling	2	24772110	24828889	seq56.78kb_op36.72+19.59)
Gap_filling	3	4813731	4854252	seq40.52kb_op34.92+16.72+22.32kb gap underestimated
Gap_filling	3	13448303	13448303	38_fragments_totalling_247.5kb_inserted gap underestimated
Gap_filling	3	16346784	16346784	7_fragmenst_totalling_107.25kb gap underestimated
Gap_filling	3	31046887	31066253	seq19.37kb_op36.77+8.95+6.94+13.98+4.90+11.84+22.94+12.40kb gap underestimated
Gap_filling	4	22031714	22064508	seq32.79kb_op36.43kb gap underestimated
Gap_filling	4	8357701	8422251	seq64.55kb_op59.03+1.24+13.35+23.54kb gap underestimated
Gap_filling	4	21856494	21862795	10.44+13.75kb_fragment_inserted gap underestimated
Gap_filling	5	7317327	7344341	seq27.01kb_op22.45+2.36+9.87kb gap underestimated
Gap_filling	5	17054988	17074781	seq19.79kb_op21.19kb gap underestimated
Gap_filling	5	21455273	21515479	seq60.21kb_op13.04+1.48+49.49kb gap underestimated
Gap_filling	5	27077168	27098510	seq21.34kb_op26.33+2.19+11.87+14.88+2.11+7.33kb gap underestimated
Gap_filling	6	10547383	10590317	seq42.93kb_op43.85kb gap overestimated
Gap_filling	7	11607358	11617617	seq10.26kb_op18.86+13.61kb
Gap_filling	8	2726085	2828128	seq102.04kb_op101.75kb gap underestimated
Gap_filling	9	13551933	13655403	seq62.18+41.29kb_op58.78+1.84+8.89+39.22kb gap underestimated
Gap_filling	9	11426110	11426110	3.83+12.72kb_fragments_inserted gap underestimated
Gap_filling	9	20282123	20282123	6.82+2.49kb_fragments_inserted gap underestimated
Gap_filling	9	20416503	20416503	22.19kb_fragment_inserted gap underestimated
Gap_filling	10	14139752	14192377	seq52.62kb_op48.36kb gap overestimated
Gap_filling	10	3973514	4065008	seq91.49kb_op23_fragments_totalling_420.22kb gap underestimated
Gap_filling	10	6934080	6945345	seq11.27kb_op7.30+3.43+3.86+3.21+7.16kb gap underestimated
Gap_filling	10	7530230	7530230	8.81+9.83+7.49+3.03+7.42+31.68kb_fragments_inserted gap underestimated
Gap_filling	10	9535498	9567386	seq31.89kb_op43.84+21.45kb gap underestimated
Gap_filling	10	11861219	11876822	seq12.37+3.24kb_op15.59+10.34+9.29+4.48+2.18+21.09+3.03kb gap underestimated
Gap_filling	11	7302337	7315940	seq13.60kb_op44.81kb gap underestimated
Gap_filling	11	8930864	8930864	9.92+8.95+8.60+23.21kb_fragments_inserted gap underestimated
Gap_filling	11	12917098	12917098	8.74+8.42+5.12+7.04+10.68+4.49+8.95+9.65+21.07kb_fragments_inserted gap underestimated
Gap_filling	11	19309786	19326941	seq17.16kb_op11.65kb gap underestimated
Gap_filling	11	27515267	27536085	seq1.92+2.02+16.87kb_op2.65+47.26+21.10kb gap underestimated
Misassembly	1	20563723	20567374	possible_inversion_seq1.54+2.11kb_op2.50+1.62kb gap underestimated
Misassembly	1	29574927	29582102	seq3.50+2.03+1.65kb_op4.66+2.63kb
Misassembly	1	14965172	15297549	~332 kb extra_sequence included
misassebly	2	2414507	2423458	8.95kb extra_sequence
Misassembly	2	29145541	29150418	possible inversion seq1.74+3.13kb_op3.29+1.39kb
Misassembly	3	18925165	18932668	extraSeq7.5 kbextra_sequence
Misassembly	4	32270439	32395356	seq11.94+112.98kb_op23.86+101.13kb
Misassembly	4	11914726	11987680	possible_inversion 47.621kb
Misassembly	4	14355676	14412965	possible_inversion37.206kb
Misassembly	4	11825094	11868629	seq43.54kb_op39.02kb extra_sequence
Misassembly	4	15086927	15246346	seq95.47+2.70+4.70+56.56kb_op89.21+41.61+4.66+2.74+16.35+7.11kb
Misassembly	4	25428497	25440909	seq12.41kb_op9.84kb extra_sequence
Misassembly	4	3339414	3380472	41.058kb extra_sequence
Misassembly	4	9506357	9534070	seq27.71kb_op24.06kb extra_sequence

Misassembly	4	16539675	16605084	probable_inverted
Misassembly	5	1316931	1345408	probable_inverted
Misassembly	5	9738900	9760041	seq8.90+1.86+10.38kb_op1.85+11.94+8.41kb
Misassembly	5	29799734	29874159	seq27.20+7.68+23.96+10.17+5.41kb_op8.72+31.41+7.71+37.27kb
Misassembly	5	1934306	1987240	seq52.93kb_op47.37kb extra_sequence
Misassembly	5	12243720	12326760	84.04 kb frank miasssemblies
Misassembly	6	47968	89682	5.34 kb extra_sequence included
Misassembly	6	22847416	22901876	seq54.46kb_op36.91+4.95+4.88kb
Misassembly	7	22783380	22802607	seq19.23kb_op16.82kb**
Misassembly	7	23772413	23777846	5.43 kb extra_sequence included
Misassembly	8	12494258	12512179	seq11.30+6.62kb_op4.00+9.18+5.50kb
Misassembly	8	26519107	26569213	seq12.86+17.37+8.12+11.76kb_op11.24+8.31+12.98+17.22kb
Misassembly	9	10681193	10689720	seq7.38+1.14kb_op6.10+6.29kb
Misassembly	9	22956314	23005298	seq13.67+35.31kb_op35.89+14.12kb, inverted
Misassembly	10	4859261	4868163	8.90 kb extra_sequence included
Misassembly	11	6753300	6792097	seq2.48+13.80+11.47+7.18+3.86kb_op6.28+6.96+11.61+15.88kb
Misassembly	11	11044666	11062927	seq18.26kb_op16.05kb**
Misassembly	11	11194620	11196755	three SwaI sites are missing in seq
Misassembly	11	11199731	11332405	seq:49.12+10.77+25.15+0.44+7.40+10.83+7.37+22.03 op:22.51+6.80+6.82+12.68+9.18+8.71+14.25+8.68+9.32+6.01+2.91+18.97
Misassembly	11	12022965	12036003	seq13.04kb_op10.81kb**
Misassembly	11	19298056	19326941	seq4.91+8.65+3.08+17.16_op11.65_extra sequence included
Misassembly	12	17147224	17179791	seq2.66+10.35+1.81+12.96+4.79kb_op1.79+13.83+3.05+10.59kb
Misassembly	12	13633431	13651311	seq17.88kb_op15.29kb**
Misassembly	12	15532357	15547947	seq15.59kb_op12.87kb**
Missing_cut_on_optical_map	1	1792940	1792940	
Missing_cut_on_optical_map	1	5475837	5475837	
Missing_cut_on_optical_map	1	7598120	7598120	
Missing_cut_on_optical_map	1	11051054	11051054	
Missing_cut_on_optical_map	1	11357184	11357184	
Missing_cut_on_optical_map	1	11420431	11420431	
Missing_cut_on_optical_map	1	11940772	11940772	
Missing_cut_on_optical_map	1	12051943	12051943	
Missing_cut_on_optical_map	1	12057773	12057773	
Missing_cut_on_optical_map	1	12433828	12433828	
Missing_cut_on_optical_map	1	13213128	13213128	
Missing_cut_on_optical_map	1	13403136	13403136	
Missing_cut_on_optical_map	1	14103558	14103558	
Missing_cut_on_optical_map	1	14393486	14393486	
Missing_cut_on_optical_map	1	14502511	14502511	
Missing_cut_on_optical_map	1	14532810	14532810	
Missing_cut_on_optical_map	1	17715005	17715005	
Missing_cut_on_optical_map	1	18248974	18248974	
Missing_cut_on_optical_map	1	19200376	19200376	
Missing_cut_on_optical_map	1	20066298	20066298	
Missing_cut_on_optical_map	1	20148981	20148981	
Missing_cut_on_optical_map	1	20539134	20539134	
Missing_cut_on_optical_map	1	21654005	21654005	
Missing_cut_on_optical_map	1	22392417	22392417	
Missing_cut_on_optical_map	1	22650807	22650807	
Missing_cut_on_optical_map	1	22656608	22656608	
Missing_cut_on_optical_map	1	22661646	22661646	
Missing_cut_on_optical_map	1	24500606	24500606	
Missing_cut_on_optical_map	1	24965405	24965405	
Missing_cut_on_optical_map	1	28925873	28925873	
Missing_cut_on_optical_map	1	29057021	29057021	
Missing_cut_on_optical_map	1	30653463	30653463	
Missing_cut_on_optical_map	1	33317555	33317555	
Missing_cut_on_optical_map	1	35040090	35040090	
Missing_cut_on_optical_map	1	39001492	39001492	
Missing_cut_on_optical_map	1	41614525	41614525	
Missing_cut_on_optical_map	1	17364398	17364398	
Missing_cut_on_optical_map	1	20066298	20066298	
Missing_cut_on_optical_map	1	4608143	4608143	
Missing_cut_on_optical_map	1	34681679	34681679	
Missing_cut_on_optical_map	1	34049907	34049907	
Missing_cut_on_optical_map	1	29057021	29057021	
Missing_cut_on_optical_map	1	39001492	39001492	

Missing_cut_on_optical_map	1	17506333	17506333
Missing_cut_on_optical_map	1	42418318	42418318
Missing_cut_on_optical_map	1	19680308	19680308
Missing_cut_on_optical_map	1	33317555	33317555
Missing_cut_on_optical_map	2	632075	632075
Missing_cut_on_optical_map	2	2050591	2050591
Missing_cut_on_optical_map	2	2417390	2417390
Missing_cut_on_optical_map	2	2419649	2419649
Missing_cut_on_optical_map	2	4170886	4170886
Missing_cut_on_optical_map	2	4236798	4236798
Missing_cut_on_optical_map	2	7002994	7002994
Missing_cut_on_optical_map	2	7101628	7101628
Missing_cut_on_optical_map	2	7853257	7853257
Missing_cut_on_optical_map	2	9295030	9295030
Missing_cut_on_optical_map	2	9442103	9442103
Missing_cut_on_optical_map	2	9659377	9659377
Missing_cut_on_optical_map	2	9679264	9679264
Missing_cut_on_optical_map	2	9697778	9697778
Missing_cut_on_optical_map	2	14611688	14611688
Missing_cut_on_optical_map	2	15688607	15688607
Missing_cut_on_optical_map	2	21705813	21705813
Missing_cut_on_optical_map	2	21940781	21940781
Missing_cut_on_optical_map	2	23483075	23483075
Missing_cut_on_optical_map	2	23487292	23487292
Missing_cut_on_optical_map	2	25064160	25064160
Missing_cut_on_optical_map	2	27533015	27533015
Missing_cut_on_optical_map	2	29853201	29853201
Missing_cut_on_optical_map	2	30782455	30782455
Missing_cut_on_optical_map	2	30883169	30883169
Missing_cut_on_optical_map	2	31362958	31362958
Missing_cut_on_optical_map	2	35876619	35876619
Missing_cut_on_optical_map	2	34624084	34624084
Missing_cut_on_optical_map	2	5675023	5675023
Missing_cut_on_optical_map	2	26748391	26748391
Missing_cut_on_optical_map	2	11652345	11652345
Missing_cut_on_optical_map	2	16729390	16729390
Missing_cut_on_optical_map	2	12204638	12204638
Missing_cut_on_optical_map	3	816633	816633
Missing_cut_on_optical_map	3	2283995	2283995
Missing_cut_on_optical_map	3	3161514	3161514
Missing_cut_on_optical_map	3	4525841	4525841
Missing_cut_on_optical_map	3	5873810	5873810
Missing_cut_on_optical_map	3	10962437	10962437
Missing_cut_on_optical_map	3	11431189	11431189
Missing_cut_on_optical_map	3	13401186	13401186
Missing_cut_on_optical_map	3	13405806	13405806
Missing_cut_on_optical_map	3	13452414	13452414
Missing_cut_on_optical_map	3	13532142	13532142
Missing_cut_on_optical_map	3	13542643	13542643
Missing_cut_on_optical_map	3	13603525	13603525
Missing_cut_on_optical_map	3	14029582	14029582
Missing_cut_on_optical_map	3	14082243	14082243
Missing_cut_on_optical_map	3	15170660	15170660
Missing_cut_on_optical_map	3	15351563	15351563
Missing_cut_on_optical_map	3	16121301	16121301
Missing_cut_on_optical_map	3	16187499	16187499
Missing_cut_on_optical_map	3	17932782	17932782
Missing_cut_on_optical_map	3	20795101	20795101
Missing_cut_on_optical_map	3	20910135	20910135
Missing_cut_on_optical_map	3	21368109	21368109
Missing_cut_on_optical_map	3	23175050	23175050
Missing_cut_on_optical_map	3	23252446	23252446
Missing_cut_on_optical_map	3	24224004	24224004
Missing_cut_on_optical_map	3	24279030	24279030
Missing_cut_on_optical_map	3	25186508	25186508
Missing_cut_on_optical_map	3	25391668	25391668
Missing_cut_on_optical_map	3	27275582	27275582

Missing_cut_on_optical_map	3	29329311	29329311
Missing_cut_on_optical_map	3	29624841	29624841
Missing_cut_on_optical_map	3	29762515	29762515
Missing_cut_on_optical_map	3	32250216	32250216
Missing_cut_on_optical_map	3	32646914	32646914
Missing_cut_on_optical_map	3	33159414	33159414
Missing_cut_on_optical_map	3	34714484	34714484
Missing_cut_on_optical_map	3	35391729	35391729
Missing_cut_on_optical_map	3	35453597	35453597
Missing_cut_on_optical_map	3	13365597	13365597
Missing_cut_on_optical_map	3	4945887	4945887
Missing_cut_on_optical_map	3	25039340	25039340
Missing_cut_on_optical_map	3	18859654	18859654
Missing_cut_on_optical_map	3	19073674	19073674
Missing_cut_on_optical_map	3	6662348	6662348
Missing_cut_on_optical_map	4	1900119	1900119
Missing_cut_on_optical_map	4	2642561	2642561
Missing_cut_on_optical_map	4	2650887	2650887
Missing_cut_on_optical_map	4	3195353	3195353
Missing_cut_on_optical_map	4	3885971	3885971
Missing_cut_on_optical_map	4	4045275	4045275
Missing_cut_on_optical_map	4	4704296	4704296
Missing_cut_on_optical_map	4	5134446	5134446
Missing_cut_on_optical_map	4	7446955	7446955
Missing_cut_on_optical_map	4	8702109	8702109
Missing_cut_on_optical_map	4	10946766	10946766
Missing_cut_on_optical_map	4	11823557	11823557
Missing_cut_on_optical_map	4	13688486	13688486
Missing_cut_on_optical_map	4	13691205	13691205
Missing_cut_on_optical_map	4	13893687	13893687
Missing_cut_on_optical_map	4	16754158	16754158
Missing_cut_on_optical_map	4	16759556	16759556
Missing_cut_on_optical_map	4	16772609	16772609
Missing_cut_on_optical_map	4	17083682	17083682
Missing_cut_on_optical_map	4	18255622	18255622
Missing_cut_on_optical_map	4	18830651	18830651
Missing_cut_on_optical_map	4	22839333	22839333
Missing_cut_on_optical_map	4	25176423	25176423
Missing_cut_on_optical_map	4	28054097	28054097
Missing_cut_on_optical_map	4	28732883	28732883
Missing_cut_on_optical_map	4	30543520	30543520
Missing_cut_on_optical_map	4	33005349	33005349
Missing_cut_on_optical_map	4	3989974	3989974
Missing_cut_on_optical_map	4	2190789	2190789
Missing_cut_on_optical_map	4	4469682	4469682
Missing_cut_on_optical_map	4	22543317	22543317
Missing_cut_on_optical_map	4	1975599	1975599
Missing_cut_on_optical_map	4	30897641	30897641
Missing_cut_on_optical_map	4	12492067	12492067
Missing_cut_on_optical_map	4	21593949	21593949
Missing_cut_on_optical_map	4	8940994	8940994
Missing_cut_on_optical_map	5	3538017	3538017
Missing_cut_on_optical_map	5	5338096	5338096
Missing_cut_on_optical_map	5	5350283	5350283
Missing_cut_on_optical_map	5	7671014	7671014
Missing_cut_on_optical_map	5	9529715	9529715
Missing_cut_on_optical_map	5	9923626	9923626
Missing_cut_on_optical_map	5	10590997	10590997
Missing_cut_on_optical_map	5	11937608	11937608
Missing_cut_on_optical_map	5	12130937	12130937
Missing_cut_on_optical_map	5	12234058	12234058
Missing_cut_on_optical_map	5	14899886	14899886
Missing_cut_on_optical_map	5	15255570	15255570
Missing_cut_on_optical_map	5	15378934	15378934
Missing_cut_on_optical_map	5	15382827	15382827
Missing_cut_on_optical_map	5	15823169	15823169
Missing_cut_on_optical_map	5	17853986	17853986

Missing_cut_on_optical_map	5	18334833	18334833
Missing_cut_on_optical_map	5	18438095	18438095
Missing_cut_on_optical_map	5	18472960	18472960
Missing_cut_on_optical_map	5	18564190	18564190
Missing_cut_on_optical_map	5	18666560	18666560
Missing_cut_on_optical_map	5	18986533	18986533
Missing_cut_on_optical_map	5	19557639	19557639
Missing_cut_on_optical_map	5	19737646	19737646
Missing_cut_on_optical_map	5	19967881	19967881
Missing_cut_on_optical_map	5	23119812	23119812
Missing_cut_on_optical_map	5	23149406	23149406
Missing_cut_on_optical_map	5	23763368	23763368
Missing_cut_on_optical_map	5	24260998	24260998
Missing_cut_on_optical_map	5	25289760	25289760
Missing_cut_on_optical_map	5	25294940	25294940
Missing_cut_on_optical_map	5	27868071	27868071
Missing_cut_on_optical_map	5	28798837	28798837
Missing_cut_on_optical_map	5	28821445	28821445
Missing_cut_on_optical_map	6	1150545	1150545
Missing_cut_on_optical_map	6	1939720	1939720
Missing_cut_on_optical_map	6	3481522	3481522
Missing_cut_on_optical_map	6	3524478	3524478
Missing_cut_on_optical_map	6	3750043	3750043
Missing_cut_on_optical_map	6	4436615	4436615
Missing_cut_on_optical_map	6	4445741	4445741
Missing_cut_on_optical_map	6	6052531	6052531
Missing_cut_on_optical_map	6	8401656	8401656
Missing_cut_on_optical_map	6	8474669	8474669
Missing_cut_on_optical_map	6	8502271	8502271
Missing_cut_on_optical_map	6	8864853	8864853
Missing_cut_on_optical_map	6	9055006	9055006
Missing_cut_on_optical_map	6	9386676	9386676
Missing_cut_on_optical_map	6	12097528	12097528
Missing_cut_on_optical_map	6	12688209	12688209
Missing_cut_on_optical_map	6	13021486	13021486
Missing_cut_on_optical_map	6	15197215	15197215
Missing_cut_on_optical_map	6	16481105	16481105
Missing_cut_on_optical_map	6	17452172	17452172
Missing_cut_on_optical_map	6	18097792	18097792
Missing_cut_on_optical_map	6	18212063	18212063
Missing_cut_on_optical_map	6	19242612	19242612
Missing_cut_on_optical_map	6	20877788	20877788
Missing_cut_on_optical_map	6	23393299	23393299
Missing_cut_on_optical_map	6	23625116	23625116
Missing_cut_on_optical_map	6	25061099	25061099
Missing_cut_on_optical_map	6	26415892	26415892
Missing_cut_on_optical_map	6	26961341	26961341
Missing_cut_on_optical_map	6	27882847	27882847
Missing_cut_on_optical_map	6	29691941	29691941
Missing_cut_on_optical_map	6	11637520	11637520
Missing_cut_on_optical_map	6	18607587	18607587
Missing_cut_on_optical_map	6	5153502	5153502
Missing_cut_on_optical_map	6	28227295	28227295
Missing_cut_on_optical_map	6	20246697	20246697
Missing_cut_on_optical_map	6	19701570	19701570
Missing_cut_on_optical_map	6	1230828	1230828
Missing_cut_on_optical_map	6	7350587	7350587
Missing_cut_on_optical_map	7	413524	413524
Missing_cut_on_optical_map	7	499061	499061
Missing_cut_on_optical_map	7	636814	636814
Missing_cut_on_optical_map	7	1868467	1868467
Missing_cut_on_optical_map	7	3943367	3943367
Missing_cut_on_optical_map	7	4249114	4249114
Missing_cut_on_optical_map	7	4813412	4813412
Missing_cut_on_optical_map	7	5969054	5969054
Missing_cut_on_optical_map	7	6585161	6585161
Missing_cut_on_optical_map	7	6609433	6609433

Missing_cut_on_optical_map	7	8523248	8523248
Missing_cut_on_optical_map	7	8553139	8553139
Missing_cut_on_optical_map	7	9582141	9582141
Missing_cut_on_optical_map	7	10080617	10080617
Missing_cut_on_optical_map	7	10085690	10085690
Missing_cut_on_optical_map	7	10495046	10495046
Missing_cut_on_optical_map	7	10501543	10501543
Missing_cut_on_optical_map	7	12995140	12995140
Missing_cut_on_optical_map	7	13272150	13272150
Missing_cut_on_optical_map	7	13721390	13721390
Missing_cut_on_optical_map	7	14107146	14107146
Missing_cut_on_optical_map	7	14178733	14178733
Missing_cut_on_optical_map	7	14309751	14309751
Missing_cut_on_optical_map	7	14988550	14988550
Missing_cut_on_optical_map	7	15416199	15416199
Missing_cut_on_optical_map	7	15418418	15418418
Missing_cut_on_optical_map	7	15953937	15953937
Missing_cut_on_optical_map	7	16008884	16008884
Missing_cut_on_optical_map	7	17218805	17218805
Missing_cut_on_optical_map	7	18975510	18975510
Missing_cut_on_optical_map	7	19835830	19835830
Missing_cut_on_optical_map	7	20583378	20583378
Missing_cut_on_optical_map	7	21473206	21473206
Missing_cut_on_optical_map	7	21960555	21960555
Missing_cut_on_optical_map	7	24472482	24472482
Missing_cut_on_optical_map	7	25783607	25783607
Missing_cut_on_optical_map	7	26196215	26196215
Missing_cut_on_optical_map	7	26199126	26199126
Missing_cut_on_optical_map	7	26749127	26749127
Missing_cut_on_optical_map	7	28195050	28195050
Missing_cut_on_optical_map	7	28949936	28949936
Missing_cut_on_optical_map	7	29065522	29065522
Missing_cut_on_optical_map	7	11302963	11302963
Missing_cut_on_optical_map	7	6152610	6152610
Missing_cut_on_optical_map	7	8612696	8612696
Missing_cut_on_optical_map	7	14253334	14253334
Missing_cut_on_optical_map	7	19270093	19270093
Missing_cut_on_optical_map	7	10910291	10910291
Missing_cut_on_optical_map	8	820822	820822
Missing_cut_on_optical_map	8	829810	829810
Missing_cut_on_optical_map	8	1669840	1669840
Missing_cut_on_optical_map	8	3171748	3171748
Missing_cut_on_optical_map	8	6439510	6439510
Missing_cut_on_optical_map	8	6583484	6583484
Missing_cut_on_optical_map	8	6668025	6668025
Missing_cut_on_optical_map	8	6873680	6873680
Missing_cut_on_optical_map	8	6881658	6881658
Missing_cut_on_optical_map	8	6885483	6885483
Missing_cut_on_optical_map	8	6949035	6949035
Missing_cut_on_optical_map	8	6953340	6953340
Missing_cut_on_optical_map	8	7938628	7938628
Missing_cut_on_optical_map	8	8519746	8519746
Missing_cut_on_optical_map	8	8802190	8802190
Missing_cut_on_optical_map	8	9515920	9515920
Missing_cut_on_optical_map	8	10634817	10634817
Missing_cut_on_optical_map	8	11004158	11004158
Missing_cut_on_optical_map	8	11107476	11107476
Missing_cut_on_optical_map	8	12426608	12426608
Missing_cut_on_optical_map	8	12434181	12434181
Missing_cut_on_optical_map	8	12534254	12534254
Missing_cut_on_optical_map	8	12536166	12536166
Missing_cut_on_optical_map	8	14812210	14812210
Missing_cut_on_optical_map	8	15049141	15049141
Missing_cut_on_optical_map	8	15898897	15898897
Missing_cut_on_optical_map	8	15901602	15901602
Missing_cut_on_optical_map	8	16373033	16373033
Missing_cut_on_optical_map	8	16756298	16756298

Missing_cut_on_optical_map	8	20613509	20613509
Missing_cut_on_optical_map	8	20616478	20616478
Missing_cut_on_optical_map	8	26312725	26312725
Missing_cut_on_optical_map	8	27093344	27093344
Missing_cut_on_optical_map	8	13698170	13698170
Missing_cut_on_optical_map	8	21349611	21349611
Missing_cut_on_optical_map	8	4967916	4967916
Missing_cut_on_optical_map	8	2609027	2609027
Missing_cut_on_optical_map	9	57403	57403
Missing_cut_on_optical_map	9	1105762	1105762
Missing_cut_on_optical_map	9	2971718	2971718
Missing_cut_on_optical_map	9	3172051	3172051
Missing_cut_on_optical_map	9	4070803	4070803
Missing_cut_on_optical_map	9	5071545	5071545
Missing_cut_on_optical_map	9	5128216	5128216
Missing_cut_on_optical_map	9	5740211	5740211
Missing_cut_on_optical_map	9	5747082	5747082
Missing_cut_on_optical_map	9	6930129	6930129
Missing_cut_on_optical_map	9	7056325	7056325
Missing_cut_on_optical_map	9	7633753	7633753
Missing_cut_on_optical_map	9	8145758	8145758
Missing_cut_on_optical_map	9	8212037	8212037
Missing_cut_on_optical_map	9	8268644	8268644
Missing_cut_on_optical_map	9	8320961	8320961
Missing_cut_on_optical_map	9	8325939	8325939
Missing_cut_on_optical_map	9	9547970	9547970
Missing_cut_on_optical_map	9	9700403	9700403
Missing_cut_on_optical_map	9	9849347	9849347
Missing_cut_on_optical_map	9	7764286	7764286
Missing_cut_on_optical_map	9	10082618	10082618
Missing_cut_on_optical_map	9	12112088	12112088
Missing_cut_on_optical_map	9	12703609	12703609
Missing_cut_on_optical_map	9	12904085	12904085
Missing_cut_on_optical_map	9	12927408	12927408
Missing_cut_on_optical_map	9	13069827	13069827
Missing_cut_on_optical_map	9	14313815	14313815
Missing_cut_on_optical_map	9	17216823	17216823
Missing_cut_on_optical_map	9	17723918	17723918
Missing_cut_on_optical_map	9	18620680	18620680
Missing_cut_on_optical_map	9	20223995	20223995
Missing_cut_on_optical_map	9	21186544	21186544
Missing_cut_on_optical_map	9	21413226	21413226
Missing_cut_on_optical_map	9	7540787	7540787
Missing_cut_on_optical_map	9	4185100	4185100
Missing_cut_on_optical_map	9	3380080	3380080
Missing_cut_on_optical_map	9	16005887	16005887
Missing_cut_on_optical_map	9	11586624	11586624
Missing_cut_on_optical_map	9	7350319	7350319
Missing_cut_on_optical_map	9	7425722	7425722
Missing_cut_on_optical_map	9	3826312	3826312
Missing_cut_on_optical_map	10	424323	424323
Missing_cut_on_optical_map	10	427030	427030
Missing_cut_on_optical_map	10	2218978	2218978
Missing_cut_on_optical_map	10	3693594	3693594
Missing_cut_on_optical_map	10	4647722	4647722
Missing_cut_on_optical_map	10	4945779	4945779
Missing_cut_on_optical_map	10	5183840	5183840
Missing_cut_on_optical_map	10	5190212	5190212
Missing_cut_on_optical_map	10	5266953	5266953
Missing_cut_on_optical_map	10	5918773	5918773
Missing_cut_on_optical_map	10	7662841	7662841
Missing_cut_on_optical_map	10	8847473	8847473
Missing_cut_on_optical_map	10	8866128	8866128
Missing_cut_on_optical_map	10	10122596	10122596
Missing_cut_on_optical_map	10	10129448	10129448
Missing_cut_on_optical_map	10	10194017	10194017
Missing_cut_on_optical_map	10	10771228	10771228

Missing_cut_on_optical_map	10	11278017	11278017
Missing_cut_on_optical_map	10	12720856	12720856
Missing_cut_on_optical_map	10	13254602	13254602
Missing_cut_on_optical_map	10	15638681	15638681
Missing_cut_on_optical_map	10	18618462	18618462
Missing_cut_on_optical_map	10	22409679	22409679
Missing_cut_on_optical_map	10	12965704	12965704
Missing_cut_on_optical_map	10	2583684	2583684
Missing_cut_on_optical_map	10	16141103	16141103
Missing_cut_on_optical_map	10	12091207	12091207
Missing_cut_on_optical_map	10	2106228	2106228
Missing_cut_on_optical_map	11	1967087	1967087
Missing_cut_on_optical_map	11	4500450	4500450
Missing_cut_on_optical_map	11	5717523	5717523
Missing_cut_on_optical_map	11	5881163	5881163
Missing_cut_on_optical_map	11	7149351	7149351
Missing_cut_on_optical_map	11	9473528	9473528
Missing_cut_on_optical_map	11	9891047	9891047
Missing_cut_on_optical_map	11	10068810	10068810
Missing_cut_on_optical_map	11	10546023	10546023
Missing_cut_on_optical_map	11	10547143	10547143
Missing_cut_on_optical_map	11	14203771	14203771
Missing_cut_on_optical_map	11	14598065	14598065
Missing_cut_on_optical_map	11	14702340	14702340
Missing_cut_on_optical_map	11	15556201	15556201
Missing_cut_on_optical_map	11	15591040	15591040
Missing_cut_on_optical_map	11	15931381	15931381
Missing_cut_on_optical_map	11	16055044	16055044
Missing_cut_on_optical_map	11	17399043	17399043
Missing_cut_on_optical_map	11	17538355	17538355
Missing_cut_on_optical_map	11	18701625	18701625
Missing_cut_on_optical_map	11	18994657	18994657
Missing_cut_on_optical_map	11	20950952	20950952
Missing_cut_on_optical_map	11	22428438	22428438
Missing_cut_on_optical_map	11	24038258	24038258
Missing_cut_on_optical_map	11	24959308	24959308
Missing_cut_on_optical_map	11	27704614	27704614
Missing_cut_on_optical_map	11	7607593	7607593
Missing_cut_on_optical_map	11	3815065	3815065
Missing_cut_on_optical_map	11	12414648	12414648
Missing_cut_on_optical_map	11	18455799	18455799
Missing_cut_on_optical_map	12	1440483	1440483
Missing_cut_on_optical_map	12	2789257	2789257
Missing_cut_on_optical_map	12	3603128	3603128
Missing_cut_on_optical_map	12	3698295	3698295
Missing_cut_on_optical_map	12	4821587	4821587
Missing_cut_on_optical_map	12	4824052	4824052
Missing_cut_on_optical_map	12	5501773	5501773
Missing_cut_on_optical_map	12	6250968	6250968
Missing_cut_on_optical_map	12	7589692	7589692
Missing_cut_on_optical_map	12	8499183	8499183
Missing_cut_on_optical_map	12	8676201	8676201
Missing_cut_on_optical_map	12	10590355	10590355
Missing_cut_on_optical_map	12	11441868	11441868
Missing_cut_on_optical_map	12	12660950	12660950
Missing_cut_on_optical_map	12	12725708	12725708
Missing_cut_on_optical_map	12	12751358	12751358
Missing_cut_on_optical_map	12	12795563	12795563
Missing_cut_on_optical_map	12	12806648	12806648
Missing_cut_on_optical_map	12	13290176	13290176
Missing_cut_on_optical_map	12	16081896	16081896
Missing_cut_on_optical_map	12	16084031	16084031
Missing_cut_on_optical_map	12	18697388	18697388
Missing_cut_on_optical_map	12	18701350	18701350
Missing_cut_on_optical_map	12	18958819	18958819
Missing_cut_on_optical_map	12	19426102	19426102
Missing_cut_on_optical_map	12	20333004	20333004

Missing_cut_on_optical_map	12	20749097	20749097
Missing_cut_on_optical_map	12	21320047	21320047
Missing_cut_on_optical_map	12	21653119	21653119
Missing_cut_on_optical_map	12	22243903	22243903
Missing_cut_on_optical_map	12	23619351	23619351
Missing_cut_on_optical_map	12	26908367	26908367
Missing_cut_on_optical_map	12	26911498	26911498
Missing_cut_on_optical_map	12	20211541	20211541
Missing_cut_on_optical_map	12	19849949	19849949
Missing_cut_on_optical_map	12	14314907	14314907
Missing_cut_on_optical_map	12	14373954	14373954
Missing_cut_on_optical_map	12	8195085	8195085

*Start=start position based on sequence; End= end position based on sequence; seq=sequence; op=optical map; Ch=chromosome. ** with less confidence
